# Identification of *TSHB* gene expression profile in Duolang sheep and its functional role in granulosa cells

**DOI:** 10.1186/s12864-026-12757-0

**Published:** 2026-05-01

**Authors:** HuiPing Sun, LeXiao Zhu, GulMuhammad Shahbaz, RuoHuai Gu, ChengLong He, ShuXin Chen, ChaoFan Wang, Feng Xing, XiangLin Yan

**Affiliations:** Key Laboratory of Tarim Animal Husbandry Science and Technology, Xinjiang Production and Construction Group, School of Animal Science and Technology, Tarim University, Alaer, 843300 China

**Keywords:** Duolang sheep, GCs, *TSHB* gene

## Abstract

**Objective:**

Thyroid-stimulating hormone beta subunit (*TSHB*) gene, a member of the glycoprotein hormone beta subunit family, has been detected in ovarian tissues and granulosa cells (GCs) of several species, suggesting a potential involvement in ovarian function. However, its molecular regulatory mechanisms and functional roles in Duolang sheep remain unclear.

**Methods:**

Clone the coding sequence of the *TSHB* gene in Duolang sheep and identify the development stage when its expression is high. Models with *TSHB* overexpression and siRNA interference were constructed to investigate their effects on the proliferation, apoptosis, and hormone secretion during the follicular phase in ovarian GCs in Duolang sheep.

**Results:**

CCK-8 assays, conducted at four distinct time points, showed that *TSHB* overexpression significantly enhanced the proliferation of GCs, accompanied by the upregulation of *Cyclin E/CDK2* and *Bcl-*2, and downregulation of Bax and Caspase3. Interference showed a trend opposite to that of overexpression (*p* < 0.05). ELISA results revealed that *TSHB* overexpression significantly reduced estrogen levels. Interference showed a trend opposite to that of overexpression (*p* < 0.05), which was consistent with decreased expression of steroidogenic acute regulatory protein and *CYP19A1*. Additionally, qPCR analysis demonstrated that *TSHB* significantly upregulated the expression of *TSHR* and *FSHR* (*p* < 0.05).

**Conclusion:**

Specifically, the above results indicate that the *TSHB* gene promotes the proliferation of GCs in Duolang sheep, providing a new perspective on their follicular development.

**Supplementary Information:**

The online version contains supplementary material available at 10.1186/s12864-026-12757-0.

## Introduction

Xinjiang has a high concentration of ethnic minorities, for many of whom mutton is a staple part of their diet. With rising living standards across the region, distinct regional preferences for mutton consumption have emerged. The combined consumption of mutton in the northwestern and northern areas of China accounts for over half of the national total, with Xinjiang having the highest consumption [1]. However, most sheep breeds in Xinjiang are characterized by late sexual maturity, delayed first estrus, and low reproductive rates. Consequently, an acute imbalance has emerged between mutton supply and demand, with pressure on the market continually increasing. To alleviate this pressure, it is important to breed sheep with earlier first estrus and higher lambing rates.In this context, Duolang sheep, a local breed indigenous to Xinjiang, China, stand out for their superior reproductive performance, characterized by early sexual maturity and high fecundity. Rams typically reach sexual maturity at 6–7 months of age, while ewes attain sexual maturity at approximately 6 months of age, with most ewes lambing within the first year. Under favorable management conditions, ewes can achieve two lambings per year or three lambings in two years, with an average flock lambing rate of 168%, a twinning rate of approximately 50%, and an annual lamb survival rate of around 150% [2]. Therefore, this breed represents a valuable genetic resource for improving reproductive efficiency and addressing the growing demand for mutton in Xinjiang.

The ovaries are the primary organs for the female reproductive system, and their primary functions include follicular development, ovulation, and hormone secretion. Follicular growth and maturation are vital components of female fertility. These structures contain oocytes and GCs, and their development involves the proliferation and differentiation of GCs [3]. Apoptosis of GCs is the primary cause of follicular atresia [4]. Although GCs play a crucial role in oocyte maturation and follicular development, the overall mechanisms by which their biological functions and molecular regulatory mechanisms specifically affect follicular development across different species have not yet been fully elucidated. In Duolang sheep, the molecular regulatory pathways underlying GC-mediated follicular development have not yet been fully elucidated. Understanding these mechanisms is essential for clarifying how GCs coordinate follicular growth and hormone secretion. For example, our previous studies have revealed that estradiol directly affects GCs in Duolang sheep via the estrogen receptor β (ERβ) to promote the first estrus [5]. Other factors, such as the *Lin28B/let-7* axis, also promote GCs proliferation and estradiol secretion (E2), playing a crucial role in regulating puberty onset [6]. Therefore, in-depth exploration of these genes and potential factors in the regulation of estrus in Duolang sheep is crucial to comprehensively understand GCs-driven follicular development.

Recent studies have shown that the *TSHB* is expressed in reproductive tissues and may play an important role at the cellular level. For example, the expression of *TSHB* and thyroid-stimulating hormone receptor (*TSHR*) has been detected in the ovaries and GCs of *Monopterus albus*, suggesting that they may exert paracrine or autocrine functions in the gonads [7]. Mutations in the *TSHB* gene cause congenital central hypothyroidism (CCH), indicating its essential role in endocrine regulation [8]. Clinical evidence suggests that thyroid dysfunction can influence gonadal development, as some children with juvenile hypothyroidism exhibit precocious puberty [9]. Using a rat model and adult-onset hypothyroidism has been shown to disrupt normal follicular development and impair fertility by promoting the apoptosis of GCs in antral follicles and inducing oxidative stress in oocytes [10]. In summary, although *TSHB* expression has been detected in ovarian GCs, similar research in the GCs of Duolang sheep is limited. Current in vitro studies mainly focus on illustrating the mechanism of action of thyroid-stimulating hormone (TSH) by detecting pathway activation; however, studies assessing the biological effects of TSH and its involvement in the functional regulation of GCs of Duolang sheep remain scarce.

In this study, we cloned the coding sequence (CDS) region of the *TSHB* gene in Duolang sheep and analyzed its molecular characteristics. Using the pcDNA3.1 vector and siRNA, we achieved targeted overexpression and interference of the *TSHB* gene and investigated its effects on granulosa cells (GCs) under in vitro conditions, with a focus on GCs survival rate and proliferation. Additionally, we analyzed the expression of *TSHR* and *FSHR* after overexpression and interference of *TSHB*, which helps to better understand the regulatory relationship between *TSHB* and these receptors. Although the expression of *TSHB* in reproductive tissues has been reported in other species, its role in the gonads and GCs of Duolang sheep remains unclear.This study aims to fill this gap by investigating the species-specific expression of *TSHB* in Duolang sheep, which may differ from that in other species. Furthermore, we explored the dual effects of *TSHB* on GC proliferation and steroidogenesis, providing new insights into its regulatory role in follicular development.

## Materials and methods

### Experimental animals and sample collection

All experimental sheep were obtained from the Animal Husbandry Experimental Station of Tarim University. Female Duolang sheep of similar body sizes were selected and reared under the same environmental conditions. Estrus was detected in Duolang sheep using the ram teasing and vulvar observation methods. Ram teasing was performed twice daily (at 10:00 and 18:00) starting from when the sheep were three months old. Sheep were selected at three stages: prepuberty (3-month-old lambs), puberty (ewes showing estrous behaviors including actively approaching rams, standing still to accept ram mounting, accompanied by vulvar mucus secretion and swelling, tail wagging, vocalization, and decreased appetite), and post-puberty (ten days after the cessation of the first estrous behaviors), with five sheep selected at each stage. Immediately after slaughter, five types of tissue samples were collected, including the hypothalamus, hypophysis, ovary, oviduct, and uterus. Due to differences in tissue size, larger tissues (hypothalamus, oviduct, and uterus) were cut into approximately 2 cm pieces, while the smaller tissues (pituitary gland and ovary) were collected and processed in their entirety without cutting. For ovarian samples specifically, both the left and right ovaries were collected from each ewe, and the entire ovaries were used without dissecting specific structures. The two ovaries from the same animal were pooled together to ensure sufficient RNA yield. which were then placed in cryopreservation tubes, stored under − 80℃ ultra-low temperature conditions for subsequent RNA extraction.

Female Duolang sheep at 45–48 h post-estrus (follicular phase) were euthanized via jugular vein exsanguination. The ovaries were rapidly collected using sterile surgical scissors, disinfected by spraying with 75% ethanol, and stored in a bottle containing pre-cooled PBS (Gibco, Grand Island, NE, USA) (pre-cooled with ice packs in a foam-insulated box). The ovaries were quickly transferred to a sterile cell culture room for subsequent operations within 4–5 h. After removing the connective tissue surrounding the ovaries, they were rinsed 3–5 times with normal saline supplemented with 3% double antibodies (penicillin-streptomycin).

### Primer design and synthesis

The *TSHB* nucleotide sequence (accession number XM_004002368.6) was retrieved from the NCBI Nucleotide database (https://www.ncbi.nlm.nih.gov). All primers used in this study were listed in Table 1. Primers were designed using Primer Premier 5.0 software (PREMIER Biosoft) and synthesized by Sangon Biotech (Shanghai) Co., Ltd., Shanghai, China.The cloned *TSHB* sequence has been deposited in GenBank under accession number PX765972.1.

**Table 1:** Primer sequences

Gene	Primer Sequence (5′→3′)	Annealing Temperature (°C)	Fragment Length (bp)	Purpose
*TSHB*	F: CATTACAACATCAGCTCAC	55.8	498	Cloning
	R: CCAGGTAAATACATTTAACC			
Q*TSHB*	F: CAGGGCATATTTGGGAAGAAAC	58	95	qPCR
	R: GTGCTTACTGCCTAACCATCAACA			
*ACTB*	F: TTCCAGCCTTCCTTCCTG	58	109	qPCR
	R: CCGTGTTGGCGTAGAGGT			
*Cyclin E*	F: GATGTCGGCTGCTTAGAAT	58	103	qPCR
	R: ACCACTGATACCCTGAAAC			
*CDK2*	F: CAGGGCATATTTGGGAAGAAAC	58	95	qPCR
	R: CCGTGTTGGCGTAGAGGT			
*Bax*	F: AACATGGAGCTGCAGAGGAT	58	208	qPCR
	R: CCAATGTCCAGCCCATGATG			
*Bcl-2*	F: TCTTTGAGTTCGGAGGGGTC	58	191	qPCR
	R: GGAGAAATCAAACAGGGGCC			
*Caspase3*	F: TGGACCCGTCGATCTGAAAA	58	195	qPCR
	R: GCGTACAAGAAGTCTGCCTC			
*STAR*	F: CAGCCCATGGAGAGGCTTTA	58	133	qPCR
	R: CAGCCAACTCGTGAGTGATG			
*CYP19A1*	F: TCATCCTGGTCACCCTTCTG	58	115	qPCR
	R: CGGTCGCTGGTCTCGTCTGG			
*TSHR*	F: CTCACTCGGGCTGACCTCTCT	58	75	qPCR
	R: TAGGATTCCCCTGATTTTCTT			
*FSHR*	F: GTTATGTCCCTCCTTGTGC	58	127	qPCR
	R: GCTTGGCTATCTTGGTGTC			

### Total RNA extraction and reverse transcription

Total RNA was extracted using TRIzol reagent (Invitrogen, Carlsbad, CA, USA) from five reproductive tissues of Duolang sheep. Separately, GCs were transfected with either a *TSHB* overexpression vector (pcDNA3.1-*TSHB* and pcDNA3.1) or *TSHB*-specific siRNAs (si-*TSHB* and si-NC).Subsequently, total RNA was extracted from the transfected cells using TRIzol reagent.RNA integrity was assessed by 1.5% agarose gel electrophoresis. RNA concentration and purity were measured using a NanoDrop 8000 spectrophotometer (NanoDrop Technologies, Wilmington, DE, USA). cDNA was synthesized using the TaKaRa Reverse Transcription Kit (TaKaRa Bio Inc., Dalian, China) for subsequent qRT-PCR analysis and PCR-based cloning.

### Cloning and sequencing of the *TSHB* gene sequence

Using the post-transcribed cDNA from the pituitary gland of Duolang sheep at Puberty as the amplification template, a 25 µL PCR reaction was assembled, containing 12.5 µL of 2× EasyTaq PCR SuperMix (+ dye) (TransGen Biotech Co., Ltd., Beijing, China), 1 µL each of forward and reverse primer (10 µM), 1 µL of cDNA template, and 9.5 µL nuclease-free water. The PCR conditions were: pre-denaturation at 95 °C for 5 min; 30 cycles of 95 °C for 30 s, 55.8 °C for 30 s, and 72 °C for 30 s; followed by a final extension at 72 °C for 15 min. The 498 bp amplicon was resolved on a 1.5% agarose gel, purified, ligated into the pMD19-T vector, and transformed into E. coli DH5α. PCR screened white colonies, and verified Sangon sequenced clones. Positive colonies (identified as white colonies in blue-white screening) were screened by colony PCR. Three independent positive clones were then selected for bidirectional Sanger sequencing (Sangon Biotech, Shanghai, China) to verify the insert sequence and exclude potential PCR-induced mutations. All three sequenced clones yielded identical sequences that fully matched the expected *TSHB* reference sequence.

### Bioinformatics analysis of the *TSHB* gene

The *TSHB* gene sequence of Duolang sheep obtained by sequencing was analyzed for its physicochemical properties and protein structure using online bioinformatics software (Table 2).

**Table 2:** Bioinformatics analytical tools

analytical tools	websites
ORF Finder	https://www.ncbi.nlm.nih.gov/orffinder/
ProtParam	https://web.expasy.org/protparam/
ProtScale	https://web.expasy.org/protscale/
TMHMM 2.0	https://services.healthtech.dtu.dk/services/TMHMM-2.0/
SignalP 6.0	https://services.healthtech.dtu.dk/services/SignalP-6.0/
PSORTⅡ	https://psort.hgc.jp/form2.html
NetPhos 3.1	https://services.healthtech.dtu.dk/services/NetPhos-3.1/
SOPMA	https://npsa-prabi.ibcp.fr/cgi-bin/npsa_automat.pl?page=npsa_sopma.html
SWISS-MODEL	https://swissmodel.expasy.org/interactive
STRING 11.0	https://cn.string-db.org

The Duolang sheep *TSHB* gene sequence was assembled and aligned, translated to amino acids, and similarity-aligned against NCBI TSHB sequences from sheep (*Ovis aries*) (XP_004002417.2), goat (*Capra hircus*) (NP_001274505.1), cattle (*Bos tauru*s) (NP_776630.1), pig (*Sus scrofa*) (NP_999533.1), cat (*Felis catus*) (XP_044889158.1), human (*Homo sapiens*) (NP_000540.2), rhesus macaque (*Macaca mulatta*) (XP_015004345.1), mouse (*Mus musculus*) (NP_001159411.1), chicken (*Gallus gallus*) (NP_990394.1), and zebrafish (*Danio rerio*) (NP_852471.1); a phylogenetic tree was constructed using MEGA 7.

### Detection of *TSHB* gene expression levels in Duolang sheep at different stages and in other tissues

To investigate the expression levels of the *TSHB* gene in different gonadal tissues of Duolang sheep during various stages of the estrous cycle, this experiment utilized the primers designed in Table 1, with *ACTB* as the endogenous control for normalization, the CFX96 Real-Time PCR Detection System (Bio-Rad, USA) was used to detect the expression levels of the *TSHB* gene in the hypothalamic-pituitary-gonadal (HPG) axis (hypothalamus, hypophysis, ovary, uterus, oviduct) of Duolang sheep before, during, and after estrus. The qPCR (15 µL) system included: 5.5 µL nuclease-free water, 0.5 µL each of forward and reverse primers, 1 µL cDNA, and 7.5 µL SYBR Green Real-time PCR Mix. The specific qPCR program was as follows: 94 °C for 30s; 40 cycles of 94 °C for 5s, 58 °C for 15s, and 72 °C for 10s. Three biological replicates were used in each experiment.

qPCR data were analyzed using the 2^−ΔΔCt^ method with *ACTB* as the internal control.

### Culture and identification of ovarian GCs

Follicles within the ovaries were incised using a sterile surgical blade, and the contents were harvested into DMEM (Gibco) medium supplemented with 10% fetal bovine serum (FBS) (ExCell Bio. Jiangsu, China), followed by centrifugation at 1,500 rpm for 5 min (Eppendorf AG, Centrifuge 5702). This resuspension-centrifugation step was repeated 3 times to obtain a purified granulosa cell pellet. The centrifuged GCs were inoculated into a T25 culture flask containing 7 mL of DMEM medium supplemented with 15% FBS and 1% penicillin-streptomycin, and then cultured at 37 °C with 5% CO₂.

GCs were identified by immunofluorescence. Cells were seeded at 1 × 10^^5^ cells/well in 24-well plates and fixed with 4% paraformaldehyde (Solarbio Science & Technology Co., Ltd., Beijing, China) for 90 min. After three washes with PBS (Gibco), 5 min each, cells were permeabilized with 0.1% Triton X-100 (Solarbio) for 10 min and blocked with 10% goat serum (Solarbio) for 30 min at room temperature. Cells were incubated overnight at 4 °C with anti-FSHR primary antibody (Proteintech Group, Inc., Wuhan, China) at a dilution of 1:200, followed by three PBS washes and a 2-h incubation at room temperature with goat anti-rabbit IgG secondary antibody (Proteintech), also at 1:200. Nuclei were stained with DAPI (Servicebio Technology Co., Ltd., Wuhan, China) for 5 min in the dark, and then mounted with an anti-fluorescence quenching agent (Servicebio). Fluorescence images were captured using a Nikon inverted fluorescence microscope (Olympus, Tokyo, Japan).

### Plasmid construction and transfection

The *TSHB* overexpression vector (pcDNA3.1-*TSHB*) and *TSHB* siRNA were synthesized by Han Heng Biotechnology (Shanghai) Co., Ltd., Shanghai, China (Table 3). GCs were seeded in 24-well plates at a density of 1 × 10^5 cells/well. Transfection was performed using Lipofectamine 3000 (Invitrogen) according to the manufacturer’s instructions.

**Table 3:** *TSHB* gene overexpression and siRNA primer sequences

Gene	Primer Sequence (5′→3′)
*TSHB*-F	caagctgtgaccggcgcctacgaattcGCCACCatgactgctatcttcc
*TSHB*-R	ACCccATCGATggACCGGTcgGGATCCgatagaaaatcccactacatag
si-*TSHB*-F	CAUACAGAGACUUCAUGUATT
si-*TSHB*-R	UACAUGAAGUCUCUGUAUGTT
si-NC-F	UUCUCCGAACGUGUCACGUTT
si-NC-R	ACGUGACACGUUCGGAGAATT

### CCK-8 assay

Cell viability and proliferation were assessed using the CCK-8 assay (Solarbio). GCs were seeded into a 96-well plate at a density of 1 × 10^4 cells/well, followed by transfection in accordance with the manufacturer’s instructions. At 12, 24, 36, and 48 h after transfection, 10 µL CCK-8 reagent was added to each well, and the plates were incubated for 2 h at 37 °C. Absorbance was measured at 450 nm using a microplate reader (Thermo Fisher Scientific). Viability was calculated relative to the control, and data (*n* = 3) are presented as mean ± SD.

### The effect of the *TSHB* gene on proliferation trend, apoptosis, steroid synthesis, and secretion of ovarian GCs in Duolang sheep

In this experiment. qPCR was used to detect key proliferation genes *Cyclin E* and Cyclin-dependent kinase 2(*CDK2*) (which are involved in cell transition from G1 phase to S phase), pro-apoptotic gene Bcl-2-associated X protein (*Bax*), anti-apoptotic gene B-cell lymphoma 2(*Bcl-2*), key apoptotic gene Cysteine-aspartic protease 3(*Caspase3*), as well as steroid-related hormone genes steroidogenic acute regulatory protein(*STAR*) and Cytochrome P450 Family 19 Subfamily A Member 1(*CYP19A1*) in GCs after *TSHB* overexpression and interference. Previous studies have found that *TSHR* and follicle-stimulating hormone receptor (*FSHR*) are closely related to *TSHB*, and the relationship between them was further verified in this experiment. The gene sequences mentioned above are shown in Table 1. Cell supernatants were collected 48 h after transfection using an ELISA kit (Solarbio), and the levels of E2 and progesterone (P4) in GCs with *TSHB* overexpression and interference were detected. The absorbance (OD) was measured at 450 nm using a microplate reader, and the concentration of the corresponding sheep hormone in the sample was calculated from the standard curve (*n* = 3).

### Western blotting

Crack the GCs using RIPA buffer (Servicebio) and centrifuge to obtain the supernatant. Protein concentration was measured by the BCA assay (Solarbio). Samples were combined with 2× SDS loading buffer (Thermo Fisher), denatured at 95 °C for 5 min, and 20 µg protein per lane was loaded. Proteins were separated using gels prepared with a one-step PAGE kit (Servicebio) and electrophoresed at 80 V for 20 min, followed by 120 V for 60 min. Proteins were transferred onto PVDF membranes at 150 mA for 70 min. Membranes were blocked for 2 h at room temperature, incubated overnight at 4 °C with primary antibodies (TSHB, β-actin, GAPDH, CDK2, Cyclin E, Bax, Bcl-2, Caspase3, STAR, CYP19A1, TSHR, FSHR) (Table 4). Washed 3 × 10 min with TBST (Solarbio), incubated with secondary antibody for 1 h at room temperature, and developed in the dark for 2 min with chromogenic substrate (TransGen). Blots were imaged on a Tanon 4000SF system and band densities quantified in ImageJ (v1.54 h). To ensure accurate normalization and to avoid potential band overlap due to similar molecular weights between certain target proteins and a single housekeeping protein, both β-actin and GAPDH were used as loading controls in Western blot analyses. The relative intensities of target protein bands were normalized to the corresponding housekeeping protein (either β-actin or GAPDH, selected based on optimal band separation). All experiments were performed in three independent biological replicates per group, and data are presented as mean ± SD.

**Table 4:** Table information of antibodies used in the experiment

Article Number	Antibodies	Dilution Ratio	Place of Origin
Ab155958	Anti-TSHB	1:5000	Abcam, Shanghai, China
60004-1-Ig	β-actin	1:10000	Proteintech, Wuhan, China
10494-1-AP	GAPDH	1:10000	Proteintech, Wuhan, China
10122-1-AP	CDK2	1:3000	Proteintech, Wuhan, China
bsm-52048R	Cyclin E	1:1000	Bioss, Beijing, China
50599-2-IG	Bax	1:5000	Proteintech, Wuhan, China
bsm-61074R	Bcl-2	1:1000	Bioss, Beijing, China
bsm-61071R	Caspase3	1:1000	Bioss, Beijing, China
bs-20387R	STAR	1:1000	Bioss, Beijing, China
16554-1-AP	CYP19A1	1:1000	Proteintech, Wuhan, China
14450-1-AP	TSHR	1:1000	Proteintech, Wuhan, China
bs-0895R	FSHR	1:1000	Bioss, Beijing, China

### Statistical analyses

Statistical analysis was performed using SPSS 26.0 software. Three biological replicates were performed for each group in RT-qPCR, Western blot and CCK8 experiments, and the data are expressed as the mean ± SD. Graphs were generated using GraphPad Prism 9.0.* *p* < 0.05 and ** *p* < 0.01 indicate statistically significant differences between groups.

## Results and analysis

### Cloning and sequence analysis of the *TSHB* gene in Duolang sheep

**Fig. 1:**
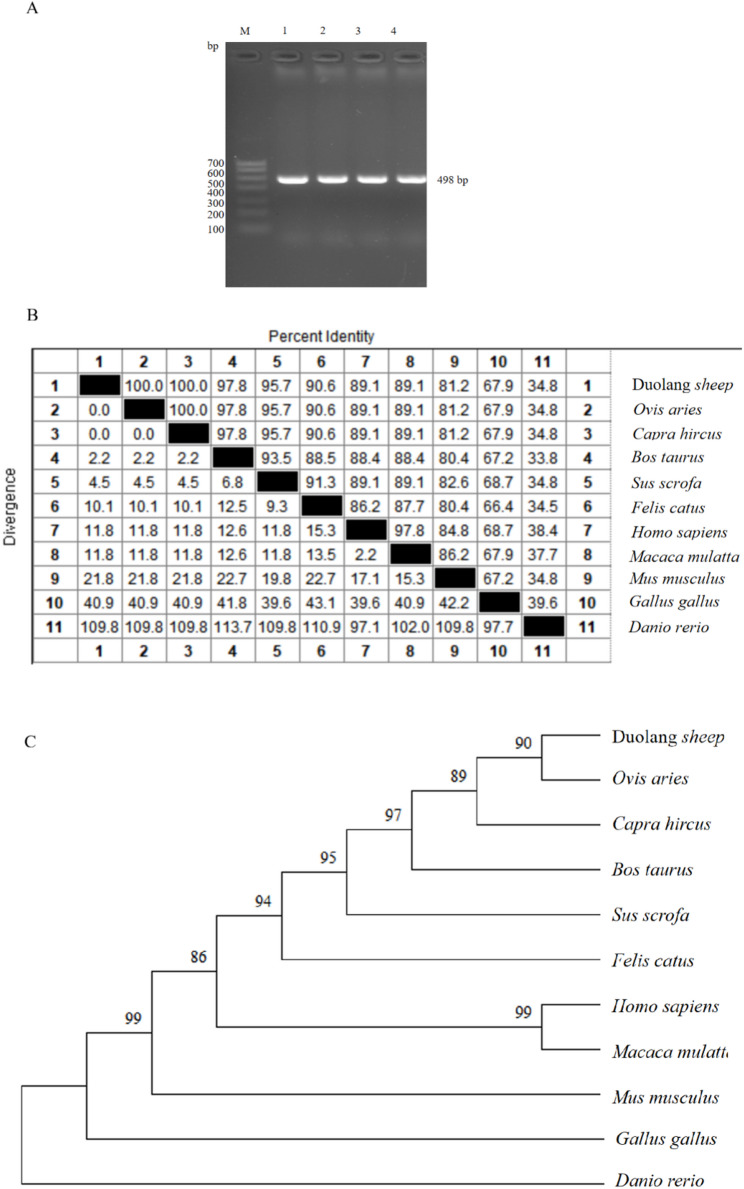
Cloning and nucleotide sequence analysis of *TSHB* in Duolang sheep. **A** Agarose gel electrophoresis detection of the cloned coding sequence of *TSHB* in Duolang sheep. **B** Comparison of nucleotide homology between the sequence obtained in this study and sequences of the *TSHB* gene from other species. **C** Neighbor-joining tree of the sequence obtained in this study and TSHB sequences identified in other species

Using the synthesized *TSHB* gene-specific primers and pituitary cDNA of Duolang sheep at puberty as the template, an amplified product with a fragment length of 498 bp was obtained (Fig. 1A). Alignment with DNAMAN and NCBI BLAST software showed that the amplified sequence exhibited high homology with the predicted sheep *TSHB* gene sequence (NCBI Accession: XM_004002368.6). After translating the amplified *TSHB* gene sequence into amino acids, we aligned the amino acid sequences with those of nine other animal species. The amino acid sequence similarities between Duolang sheep TSHB and those of *Ovis aries*, *Capra hircus*, *Bos taurus*, *Sus scrofa*, *Felis catus*, *Homo sapiens*, *Macaca mulatta*, *Mus musculus*, *Gallus gallus*, and *Danio rerio* were 100%, 100%, 97.8%, 95.7%, 90.6%, 89.1%, 89.1%, 81.2%, 67.9%, and 34.8%, respectively (Fig. 1B). Phylogenetic tree analysis revealed that the *TSHB* gene of Duolang sheep exhibited an extremely close genetic relationship with those of *Ovis aries* and *Capra hircus*, whereas it has a relatively distant genetic relationship with *Danio rerio* (Fig. 1C).

### Analysis of TSHB protein properties

**Fig. 2:**
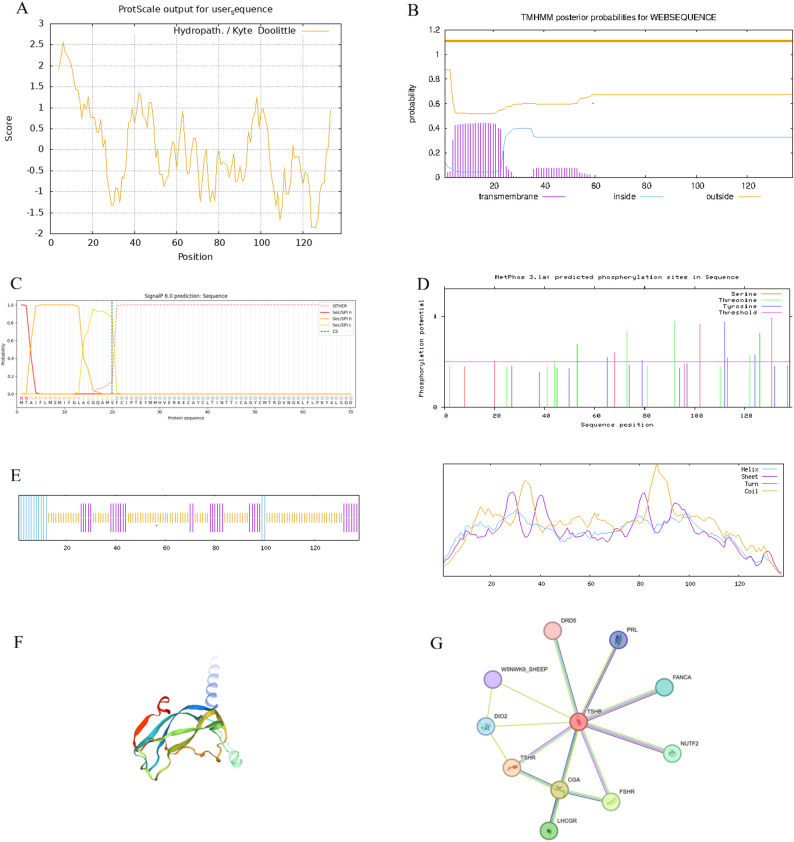
Analysis of TSHB protein properties in Duolang sheep. **A** Hydrophilicity and hydrophobicity analysis of the TSHB protein. **B** Transmembrane domain prediction of the TSHB protein. **C** Signal peptide prediction of the TSHB protein. **D** Phosphorylation site analysis of the TSHB protein. **E** Secondary structure of the TSHB protein. **F** Tertiary structure prediction of the TSHB protein. **G** Protein-protein interaction network analysis of the TSHB protein

Predictive analysis indicated that the Duolang sheep TSHB protein is 138 amino acids, with a molecular mass of 15.61 kDa and a theoretical pI of 8.20 (Table 5). Cysteine was the most abundant residue (9.4%) while tryptophan was absent. The protein contains 10 negatively charged residues and 13 positively charged residues, consistent with an overall basic character. Its aliphatic index and instability index were 66.45 and 47, respectively, and hydrophobicity profiling suggested a predominantly hydrophilic, unstable protein (Fig. 2A). No transmembrane regions were predicted (Fig. 2B), and a signal peptide cleavage site was located between residues 20 and 21 (Fig. 2C), with predicted extracellular localization (66.7% cell wall). There were 31 predicted phosphorylation sites (8 Ser, 12 Thr, 11 Tyr) (Fig. 2D). Secondary-structure prediction indicated 15 α-helices (10.9%), 31 extended strands (22.5%), and 92 random coils (66.7%) (Fig. 2E), and tertiary-structure and interaction analyses showed distributed α-helices/random coils and potential interactions with TSHR and FSHR (Fig. 2F, G).

**Table 5:** Amino acid quantity and proportion of TSHB protein in Duolang sheep

Amino Acid	Quantity (No./piece)	Proportion (%)	Amino Acid	Quantity (No./piece)	Proportion (%)
Alanine	9	6.5	Leucine	6	4.3
Arginine	4	2.9	Lysine	9	6.5
Asparagine	4	2.9	Methionine	8	5.8
Aspartic acid	5	3.6	Phenylalanine	7	5.1
Cysteine	13	9.4	Proline	7	5.1
Glutamine	3	2.2	Serine	8	5.8
Glutamic acid	5	3.6	Threonine	12	8.7
Glycine	7	5.1	Tryptophan	0	0.0
Histidine	3	2.2	Tyrosine	11	8.0
Isoleucine	10	7.2	Valine	7	5.1

### Analysis of the *TSHB* gene expression profile differences in Duolang sheep across different stages and tissues

**Fig. 3:**
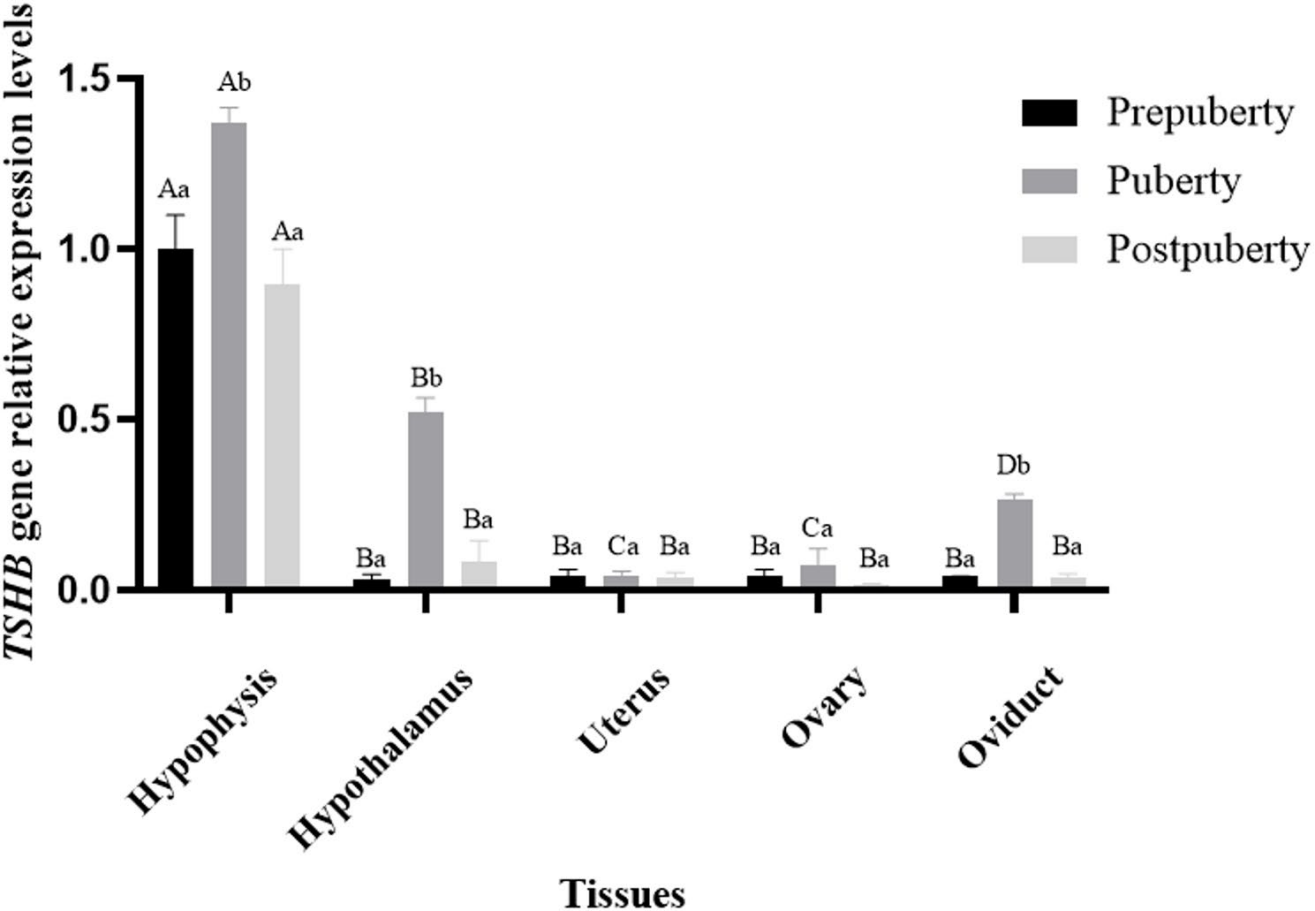
*TSHB* gene expression profile of Duolang sheep. Different capitalized letters of shoulder marks indicated significant differences in different tissues in the same period (*P* < 0.05), while the same labels indicated no significant differences (*P* < 0.05). Different lowercase letters of shoulder marks indicated significant differences in the same tissue at different periods (*P* < 0.05), while the same labels indicated no significant differences (*P* < 0.05)

*TSHB* expression in five tissues (hypothalamus, oviduct, hypophysis, ovary, and uterus) of Duolang sheep was detected using qPCR (Fig. 3). *TSHB* was expressed in all five tissues across prepuberty, puberty, and postpuberty stages. *TSHB* expression level in the hypophysis was markedly elevated compared to the other tissues (*P* < 0.05). No significant change in the *TSHB* gene expression levels in the ovarian and uterine tissues was observed (*P* > 0.05). In the hypothalamic and oviductal tissues, the expression levels of *TSHB* during puberty were significantly higher than those during prepuberty and postpuberty stages (*P* < 0.05). Hence, *TSHB* is likely important in the initiation of puberty in Duolang sheep.

### Isolation and culture of ovarian GCs from Duolang sheep

**Fig. 4:**
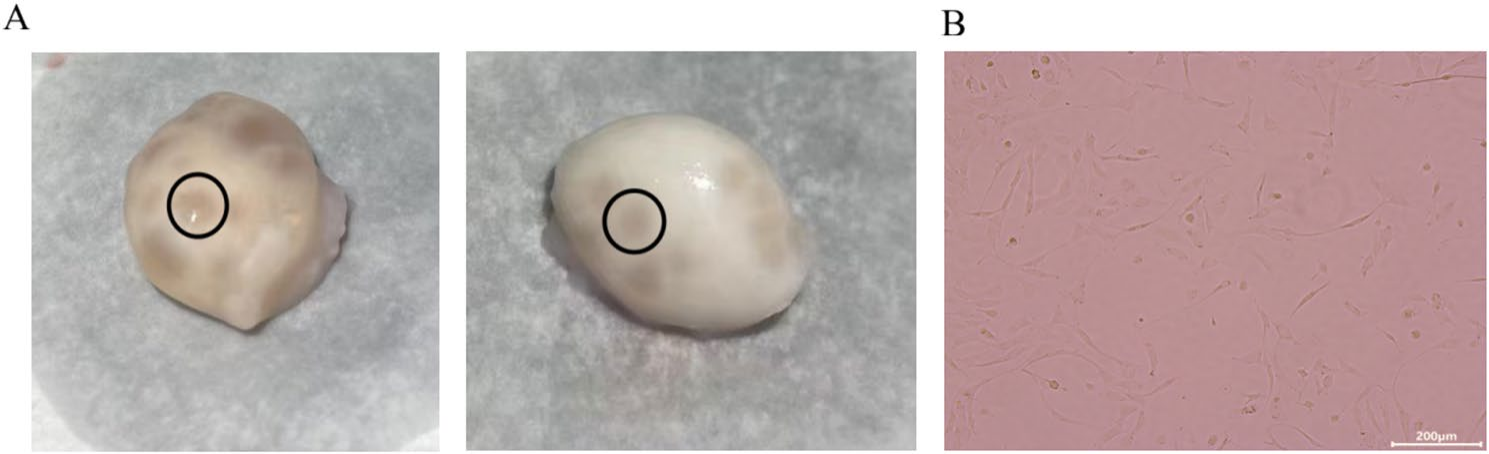
Culture of ovarian GCs from Duolang sheep. **A** Ovary of Duolang sheep; **B** Isolated GCs at the second passage

Ovarian GCs isolated by cutting the follicles (Fig. 4A) were cultured. Primary GCs were sub-cultured for 24 h. When the cells reached the second passage, they exhibited a clear morphology (short spindle-shaped) and fully covered the entire culture flask (Fig. 4B). Subsequent experiments were conducted.

### Identification of ovarian GCs from Duolang sheep

**Fig. 5:**
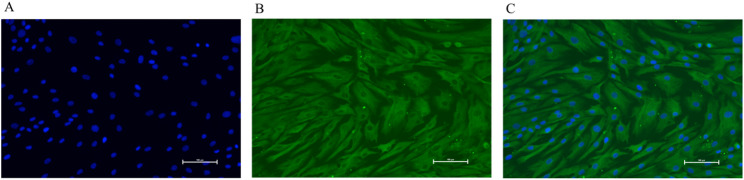
Immunofluorescence staining of GCs. **A** DAPI-stained nuclei (blue); **B** FSHR-specific immunofluorescent signal (green); **C** Merged image (DAPI + FSHR). The proportion of FSHR-positive cells exceeded 95%, indicating the high purity of isolated GCs. Scale bar = 100 μm

To confirm that the isolated cells were GCs, immunofluorescent staining was performed to detect FSHR expression (Fig. 5). It was found that the GCs isolated from the ovaries of Duolang sheep specifically expressed FSHR. The proportion of positive cells exceeded 95% (Fig. 5C), indicating that the isolated cells were GCs.

### The *TSHB* gene promotes the proliferation of GCs

**Fig. 6:**
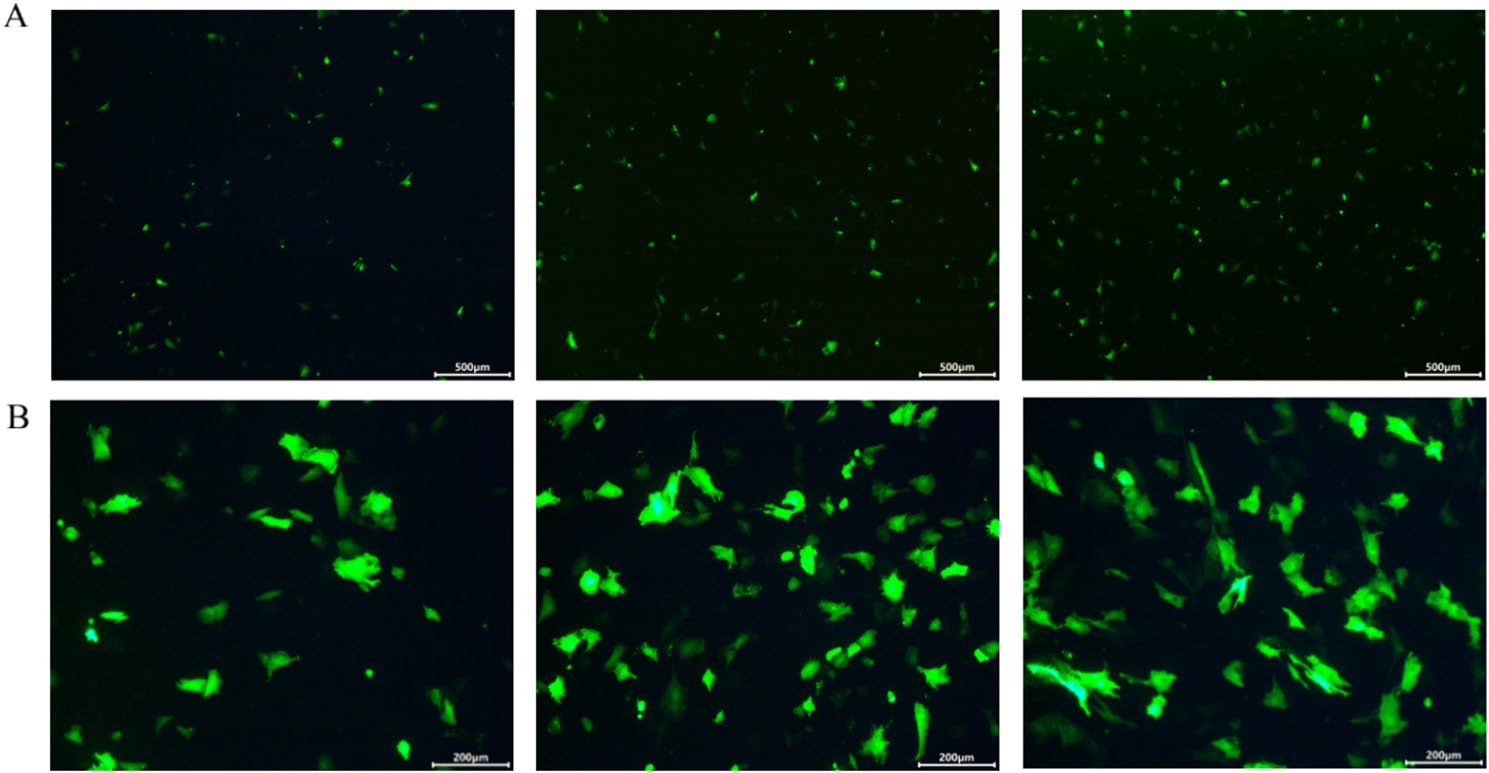
Overexpression of *TSHB* in Duolang sheep GCs. **A** Fluorescence images of empty vector transfection at 24 h, 36 h, and 48 h (Scale bar: 500 μm); **B** Fluorescence images of TSHB gene overexpression transfection at 24 h, 36 h, and 48 h (Scale bar: 200 μm)

*TSHB* expression was detected at 24, 36, and 48 h. Transfected ovarian GCs of Duolang sheep were observed under a Nikon fluorescent inverted microscope, and several green fluorescent proteins were observed (Fig. 6A, B). This indicated that the in vitro overexpression transfection was successful, and the *TSHB* gene was highly expressed in GCs.

### Effects of the *TSHB* gene on GCs proliferation and apoptosis-related genes

**Fig. 7:**
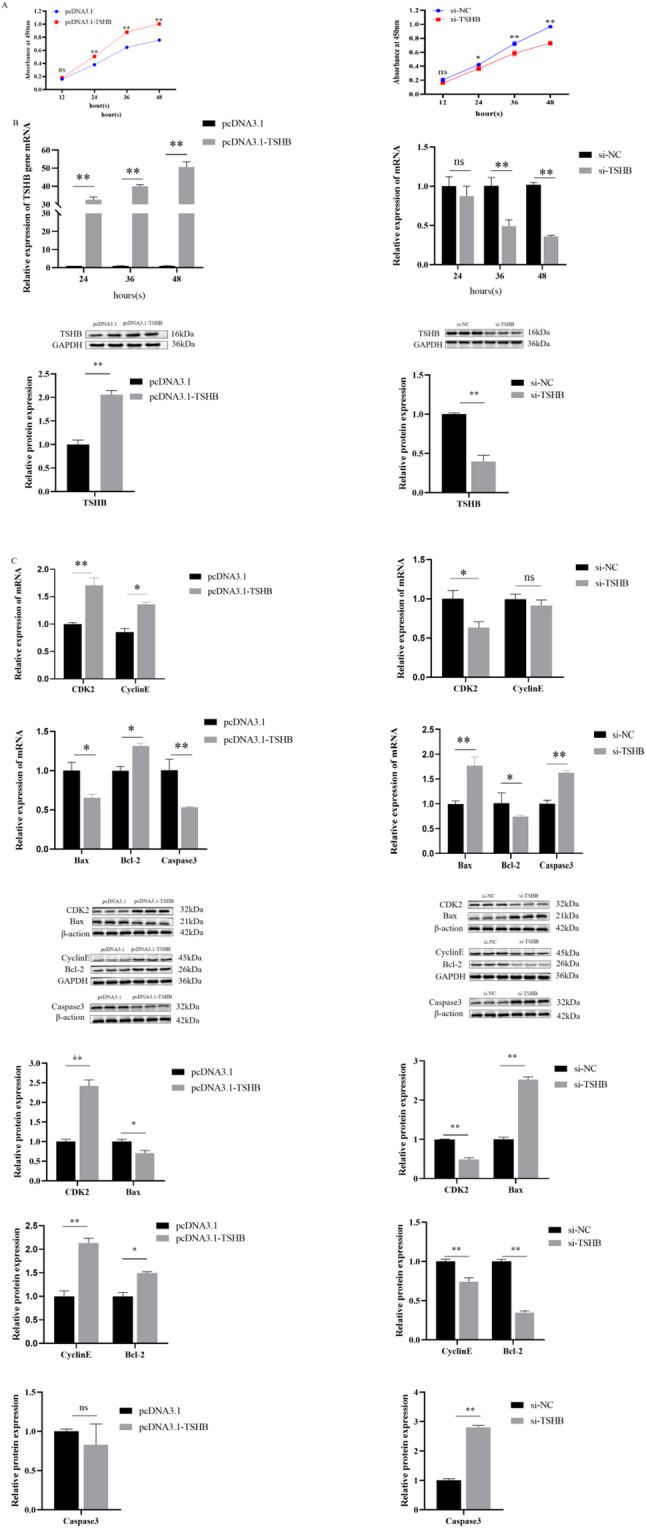
*TSHB* gene promotes GCs proliferation and inhibits cell apoptosis. **A** Effect of *TSHB* gene overexpression on ovarian GCs proliferation detected by CCK-8 assay; **B** mRNA expression levels, protein levels, and gray value analysis of *TSHB* gene after overexpression or interference; **C** mRNA expression levels, protein levels, and gray value analysis of proliferation- and apoptosis-related genes after overexpression and interference.*(*P*<0.05), **༈*P*<0.01༉ns ( not statistically significant)

The expression levels of *TSHB* showed different patterns with increasing transfection duration. After *TSHB* overexpression, the results showed that at the mRNA level, the *TSHB* gene transfection groups at 24, 36, and 48 h were significantly higher than those in the blank group (*P* < 0.01). The expression level gradually increased with time, but exhibited a decreasing trend after knockdown. The protein levels were consistent with this result, confirming its expression efficiency (Fig. 7B). With increasing time, *TSHB* gene overexpression exhibited a significant promoting effect on the proliferation of ovarian GCs (*P* < 0.01), which preliminarily demonstrated that the *TSHB* gene can promote the proliferation of ovarian GCs (Fig. 7A).

CCK-8 assay and detection of mRNA overexpression revealed that the transfection efficiency was optimal at 48 h. Therefore, this optimal period was adopted for all subsequent experiments. At 48 h after *TSHB* overexpression in ovarian GCs, qPCR analysis revealed that the mRNA levels of *Cyclin E* and *CDK2* were significantly higher than those in the empty vector control group (*P* < 0.01). Hence, the upregulation of *TSHB* may promote G1/S phase transition by enhancing the *Cyclin E/CDK2* signaling axis, thereby driving cell proliferation. In ovarian GCs with *TSHB* overexpression. The mRNA expressions of pro-apoptotic molecules *Bax* and *Caspase3* were significantly lower than those in the empty vector control group (*P* < 0.01), whereas that of anti-apoptotic factor *Bcl-2* was significantly increased (*P* < 0.01). Protein levels were consistent with the trend of mRNA expression. (Fig. 7C), suggesting that *TSHB* inhibits apoptosis by regulating the *Bcl-2*/*Bax* balance and suppressing *Caspase3* activation.

### Effects of the *TSHB* gene on estrogen and steroid hormones in GCs

**Fig. 8:**
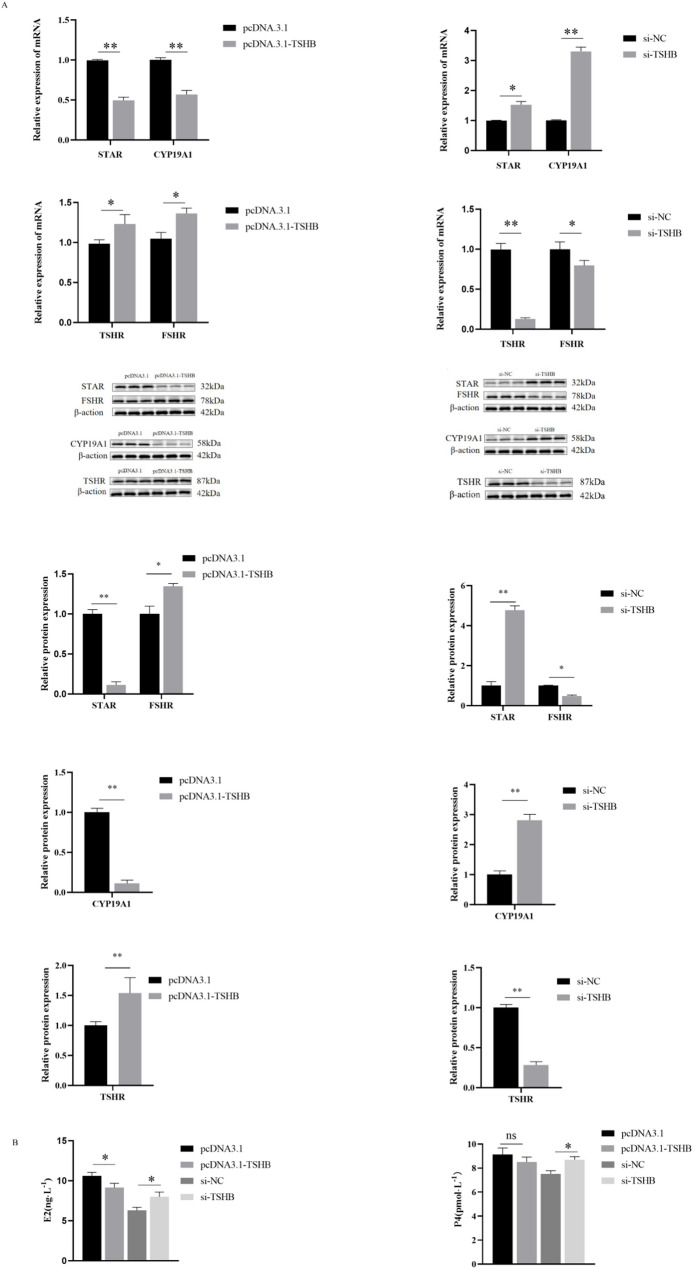
Effects of *TSHB* overexpression or interference on estrogen and steroids. **A** Effects of *TSHB* overexpression or interference on the expression of steroid secretion-related genes in ovine GCs; **B **Secretion of E2 and P4 after *TSHB* overexpression or interference

In ovarian GCs with *TSHB* gene overexpression and interference, qPCR detection of *STAR* and *CYP19A1* showed that the mRNA levels of *STAR* and *CYP19A1* were significantly lower than those in the empty vector group (*P* < 0.01) but increased after interference. The mRNA levels of the related genes, *TSHR* and *FSHR*, were significantly higher than those in the empty vector group (*P* < 0.05) and decreased after knockdown. The protein levels were consistent with the mRNA levels (Fig. 8A). Moreover, after overexpression, the secretion levels of E2 and P4 exhibited a decreasing trend (*P* < 0.05) (Fig. 8B). These results indicate that *TSHB* overexpression inhibits steroidogenesis and maintains follicle-stimulating signals by increasing gonadotropin receptor expression.

## Discussion

In 1983, Gurr JA et al. first cloned cDNA of *TSHB* in mice [11], which is a member of the glycoprotein hormone β-subunit family. It forms a heterodimeric glycoprotein composed of two subunits: a common α-subunit shared by four proteins, namely TSHB, luteinizing hormone β-subunit (LHB), follicle-stimulating hormone β-subunit (FSHB), and human chorionic gonadotropin β-subunit (hCGB), along with a hormone-specific β-subunit encoded by paralogous genes [12]. LHB, FSHB, hCGB, and TSHB of the glycoprotein hormone β-subunit family play important roles in regulating follicle growth and development [13], oocyte maturation [14], as well as testicular development and spermatogenesis [15]. The product encoded by *TSHB* forms TSH, a key glycoprotein that regulates the hypothalamic-pituitary-thyroid (HPT) axis, by combining with the common α-subunit (CGA). TSH exerts its physiological functions by acting on the TSHR [16]. TSH is released into the bloodstream and binds to TSHR located on the basolateral side of the thyroid follicular cells. TSHR is a G-protein-coupled receptor. Its activation further activates adenylate cyclase and increases intracellular cyclic adenosine monophosphate (cAMP) levels [17]. Upon binding to TSHR, TSH stimulates the synthesis and secretion of thyroid hormones, thereby regulating physiological processes, including metabolism, growth, and reproduction [7].

We cloned the coding region sequence of the *TSHB* gene in Duolang sheep and determined its molecular characteristics and expression levels in the gonadal tissues. The results showed that, *TSHB* gene was highly expressed in the pituitary glands of pubertal Duolang Sheep (*p* < 0.05). This is consistent with the previous transcriptome analysis in sheep, which found that *TSHB* exhibits different expression levels in the hypothalamus and pituitary gland under different photoperiods [18]. Hence, *TSHB* is likely a key component of the HPT axis, and its upregulated expression may be associated with the regulation of estrus or reproductive cycles. The TSHB sequence of Duolang sheep exhibited the highest homology with those of sheep and goats. Our findings confirmed that the CDS region indeed represents the *TSHB* sequence of Duolang sheep. Bioinformatics analysis revealed that the TSHB protein of Duolang Sheep is a secreted hormone subunit protein that is hydrophilic and slightly alkaline, unstable, non-transmembrane, and contains a signal peptide. Its abundant subcellular localization sites, phosphorylation sites, and potential for interaction with hormone receptors (TSHR and FSHR) provided clear clues for subsequent functional studies on *TSHB*.

Although *TSHB* is traditionally thought to be secreted exclusively in the pituitary gland and is involved in regulating the systemic thyroid axis, recent studies have shown that *TSHB* and its splice variants are also expressed in a variety of non-pituitary tissues. They can induce local cellular functions, such as proliferation, differentiation, and metabolic regulation, by activating the TSHR-cAMP pathway. A splice variant named TSH-β has been identified in bone marrow-derived macrophages of humans and mice, which can promote the proliferation and differentiation of osteoblasts [19, 20]. However, the function of *TSHB* in ovarian GCs of Duolang sheep has seldom been documented, which is why we studied the role of *TSHB* in sheep ovarian GCs.

Follicular development is a highly sophisticated process, the core of which relies on coordinated signal transmission between ovarian GCs, oocytes, cumulus cells, and gonadotropins. FSH stimulates the proliferation of GCs and the production of steroid hormones within the follicles [21]. After FSH activates FSHR, multiple signal transduction pathways and signaling molecules interact, thereby playing important roles in the proliferation and differentiation of GCs. GCs regulate cell proliferation, differentiation, steroidogenesis, and survival through the cAMP/PKA pathway [22]. GCs’ proliferation and apoptosis affect follicular development and atresia. Cell proliferation is regulated by cell cycle- and apoptosis-related genes and is a core mechanism through which cells undergo division to support organismal growth and development [23]. Recent studies have reported that the Cyclin E/CDK2 complex has a rapid activation mechanism; it initiates more quickly than Cyclin D/CDK4, enabling cells to enter the S phase rapidly, thus serving as the structural basis for cell cycle timing control [24]. The Cyclin E/CDK2 complex is a key kinase complex in the G1→S phase transition of cells. Further structural biology analyses indicated that the cyclin E/CDK2 complex possesses a rapid activation mechanism that accelerates entry into S phase [25, 26]. Bcl-2 functions as a key survival protein, preventing apoptosis by blocking mitochondrial pathways, cell apoptosis by binding to Bax, and blocking the release of cytochrome c from mitochondria [27]. Bax, a pro-apoptotic protein in the Bcl-2 family, augments mitochondrial membrane permeability. This, in turn, liberates apoptotic factors into the cytoplasm and initiates the activation of Caspase3, ultimately leading to apoptosis [28]. In this study, the CCK-8 assay results showed that *TSHB* overexpression significantly promoted GCs proliferation in Duolang sheep. Meanwhile, after *TSHB* overexpression, expression of genes involved in cell cycle progression (e.g., Cyclin E and CDK2) significantly increased in the GCs of Duolang sheep. Additionally, the expression of Bcl-2, a gene that prevents apoptosis, was elevated, whereas the expression of genes involved in programmed cell death (Bax and Caspase3) was decreased in the GCs. These findings suggest that *TSHB* may regulate GCs’ proliferation and apoptosis by modulating the expression of genes involved in cell cycle progression and apoptosis.

E2 helps reduce GC apoptosis and promotes proliferation; however, when the concentration of E2 is excessively high, it may inhibit GC proliferation [29]. The functions of P4 include slowing the follicular maturation process and inhibiting the division and apoptosis of GCs, thereby maintaining a balanced number of follicles. STAR is a rate-limiting protein in steroid synthesis and is particularly important for follicular maturation and corpus luteum formation. Its expression is rapidly upregulated after follicular rupture, and it participates in the shift in hormone synthesis pathways [30]. CYP19A1 is a key enzyme that converts androgens into estrogens; it is highly expressed in GCs and acts as a rate-limiting enzyme in estrogen synthesis, playing an instrumental role in regulating gonadotropin levels and ovarian function [31]. ELISA detected that the E2 and P4 levels in the *TSHB* overexpression group were significantly decreased, which was consistent with the suppressed mRNA expression of STAR and CYP19A1, the key factors required for steroid synthesis. This was consistent with the previous findings that high concentrations of TSH can inhibit 3β-HSD activity and steroid hormone secretion in GCs and that STAR and CYP19A1 often exhibit reverse regulation during follicular lymphangiogenesis [32]. Protein-protein interaction prediction of the TSHB protein via STRING revealed its interactions with other proteins, such as TSHR and FSHR, both of which belong to the glycoprotein hormone family. Therefore, qPCR assays were performed to evaluate the impact of *TSHB* overexpression on *TSHR* and *FSHR* expression. *TSHB* overexpression significantly increased the mRNA levels of both *TSHR* and *FSHR* in GCs. These findings suggest that *TSHB* may regulate the expression of *TSHR* and *FSHR* in GCs. Prior studies have reported that *TSHR* expression is detectable in the GCs of monkeys and humans, and that after TSH stimulation, *TSHR* expression and intracellular cAMP levels increase [33]. Furthermore, FSH signaling and hormone levels form a positive feedback loop that enhances *FSHR* expression, increasing hormone sensitivity and promoting cell proliferation. Based on these findings, we propose that *TSHB* may indirectly modulate the sensitivity of GCs to sensitivity to FSH stimulation through upregulation of *FSHR* expression. Nevertheless, this study still has limitations: the current results are mainly based on in vitro cell experiments, and subsequent confirmation of in vivo function and further in vitro studies are still required to clarify the specific mechanism of the *TSHB* gene in the development of GCs.

## Conclusion

In this study, we cloned the coding sequence of the *TSHB* gene from Duolang sheep, 498 bp. *TSHB* was detected in five gonadal tissues, peaking in the pubertal pituitary. Overexpression of *TSHB* promoted GCs proliferation, inhibited apoptosis, and downregulated *STAR* and *CYP19A1*, resulting in reduced steroidogenesis. These findings suggest that *TSHB* may exert a dual regulatory role, participating both in follicular development and in fine-tuning steroid hormone synthesis. These findings provide theoretical support for the growth and development of ovarian GCs in Duolang Sheep.

## Supplementary Information

Supplementary Material 1.

## Data Availability

The datasets used and/or analyzed during the current study are available from the corresponding author on reasonable request. Biological materials: Tissue samples and ovarian granulosa cells from Duolang sheep are preserved at the Key Laboratory of Utilization of Surrounding Tarim Livestock and Grass Resources (Tarim University) and may be requested for non-commercial research purposes. Public data resources: Reference sequences for the *TSHB* gene (NCBI Accession: XM_004002368.6) were obtained from the NCBI database (https://www.ncbi.nlm.nih.gov/). The cloned *TSHB* sequence has been deposited in GenBank under accession number PX765972.1. The deposited sequence is the trimmed version (455 bp, complete mRNA with CDS from positions 24 to 440 bp), derived from the initial 498 bp amplified product (including primer sequences). Primers: All primer sequences are provided in the Methods section.

## Abbreviations

*TSHB* gene: Thyroid-stimulating hormone beta subunit gene
GCs: Granulosa cells
ERβ: Estrogen receptor *β*
CCH: Congenital central hypothyroidism
TSH: Thyroid-stimulating hormone
CDS: Coding sequence
HPG: Hypothalamic-pituitary-gonadal
CDK2: Cyclin-dependent kinase 2
Bax: Bcl-2-associated X protein
Bcl-2: B-cell lymphoma 2
Caspase3: Cysteine-aspartic protease 3
STAR: Steroidogenic acute regulatory protein
CYP19A1: Cytochrome P450 Family 19 Subfamily A Member 1
TSHR: Thyroid-stimulating hormone receptor
FSHR: Follicle-stimulating hormone receptor
E2: Estradiol
P4: progesterone
