# Supplementary material for: Identification of TSHB gene expression profile in Duolang sheep and its functional role in granulosa cells
Source: BMC Genomics. 2026 May 1;27:427. doi: 10.1186/s12864-026-12757-0 (PMC13134140; doi:10.1186/s12864-026-12757-0)
Supplement: Supplementary file 1 — Supplementary Material 1. [file 12864_2026_12757_MOESM1_ESM.pdf]

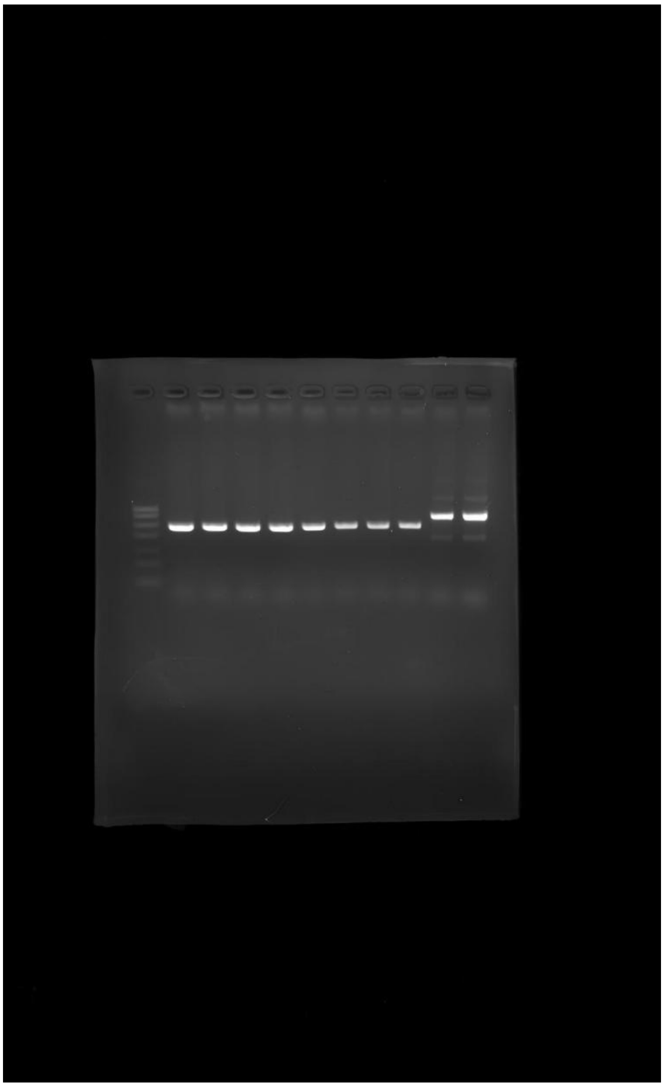

supplementary Figure1：

Agarose gel electrophoresis detection of the cloned coding sequence of *TSHB* in Duolang sheep. The gel in question was run together with samples from another experiment, so the two right-most lanes contain material that is unrelated to the current study.

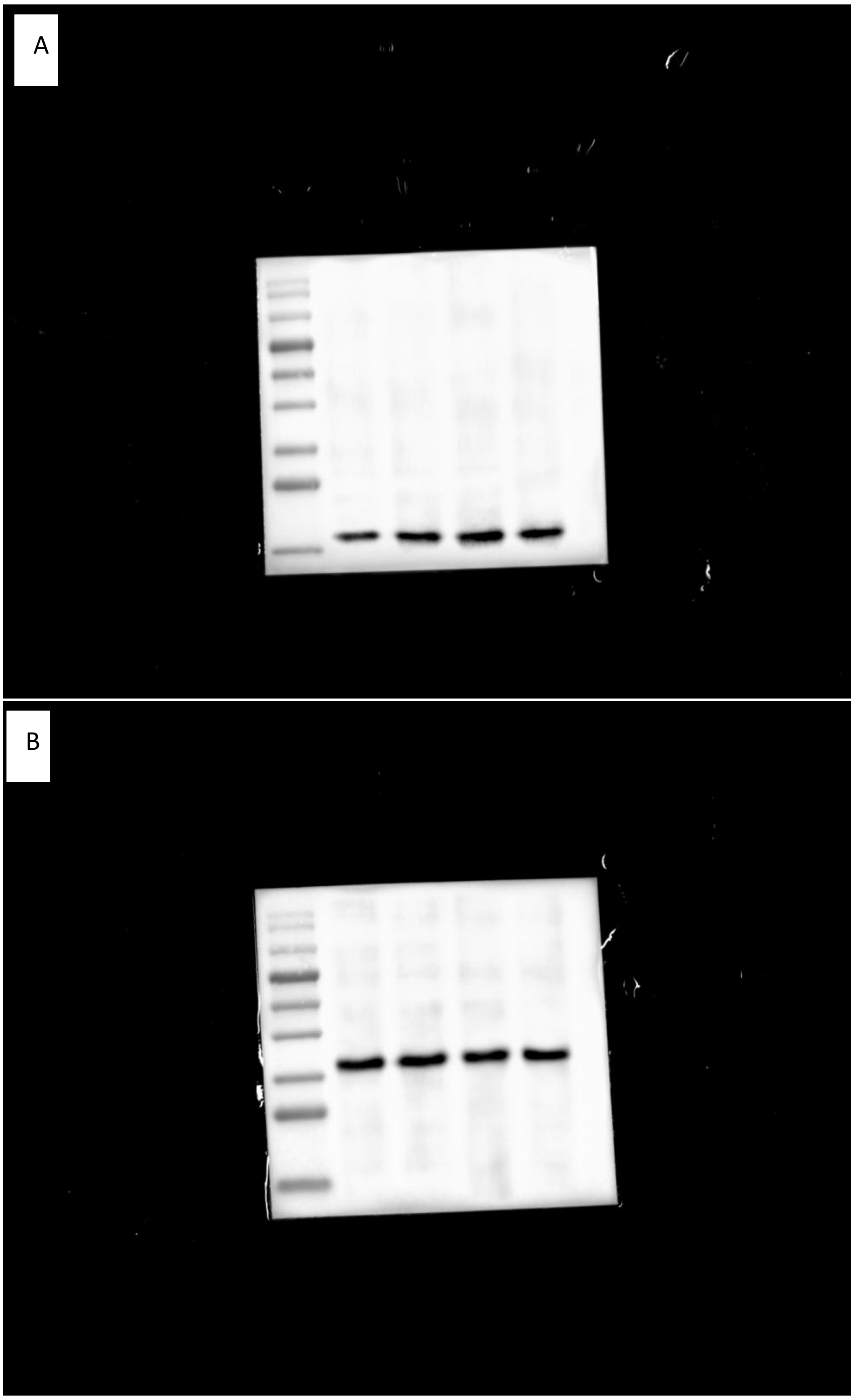

supplementary Figure2：

Figure 2A: Western blot detection of TSHB protein following overexpression.

Figure 2B: Western blot detection of GAPDH as a housekeeping protein.

A

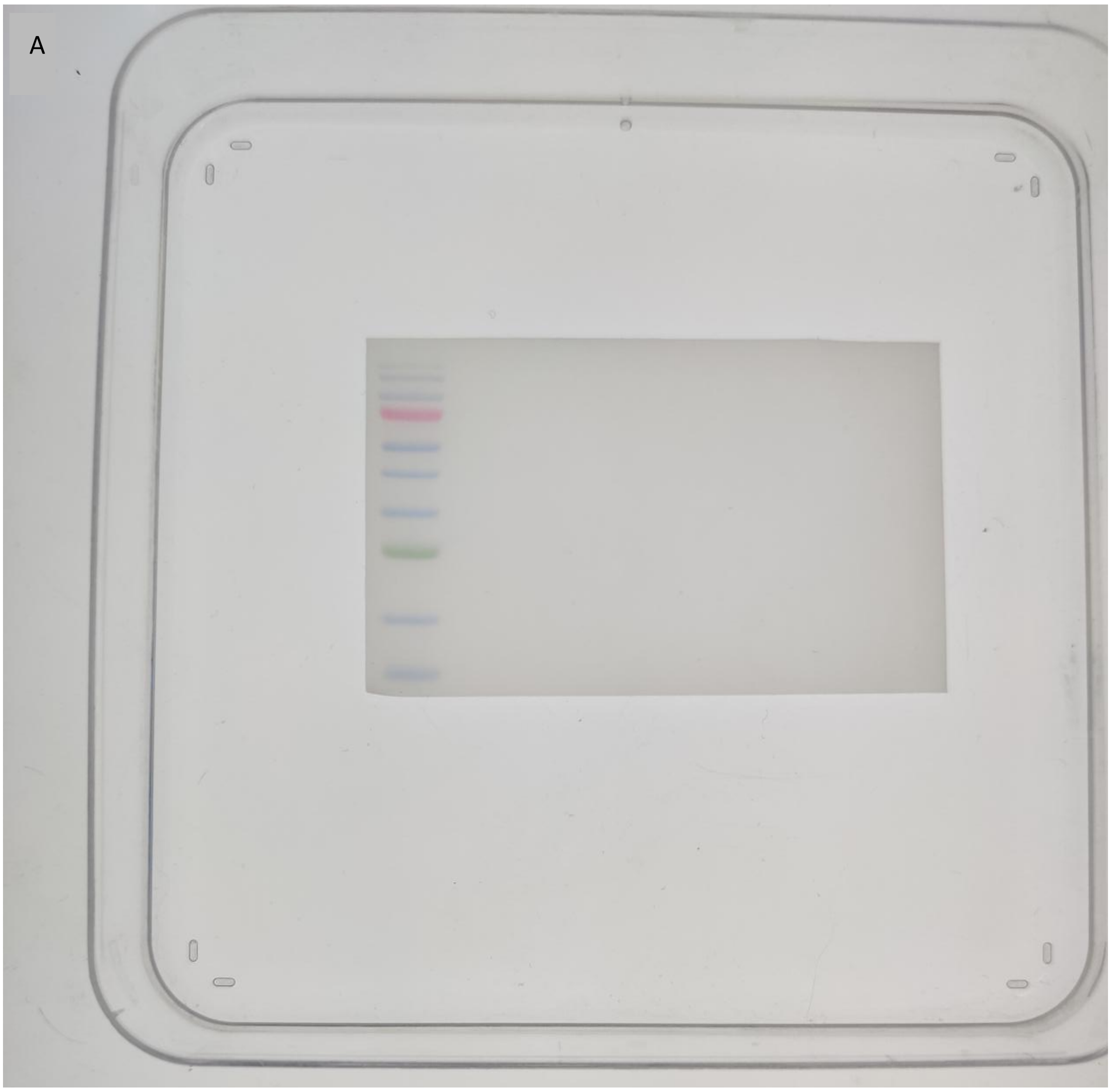

B

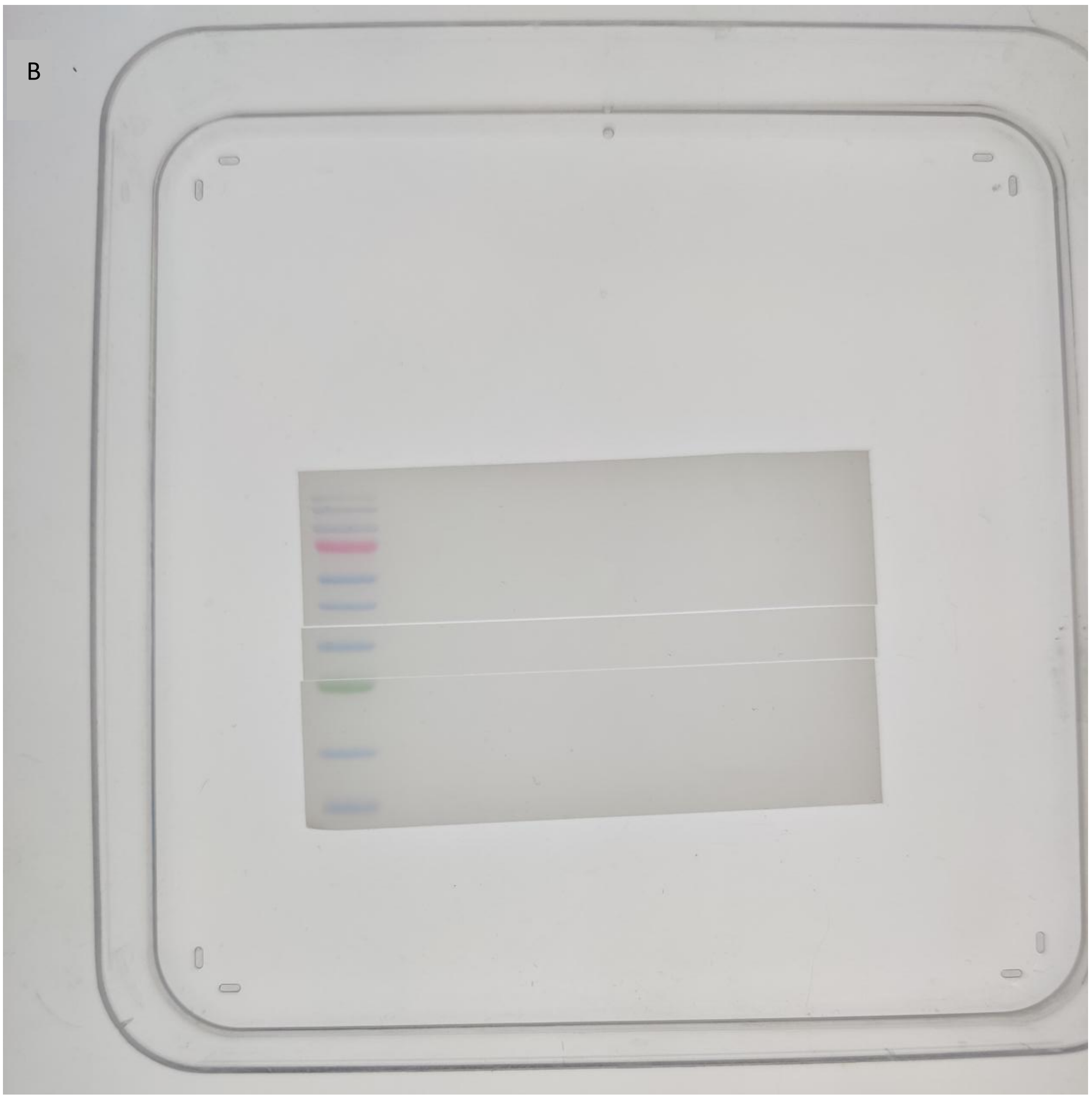

C

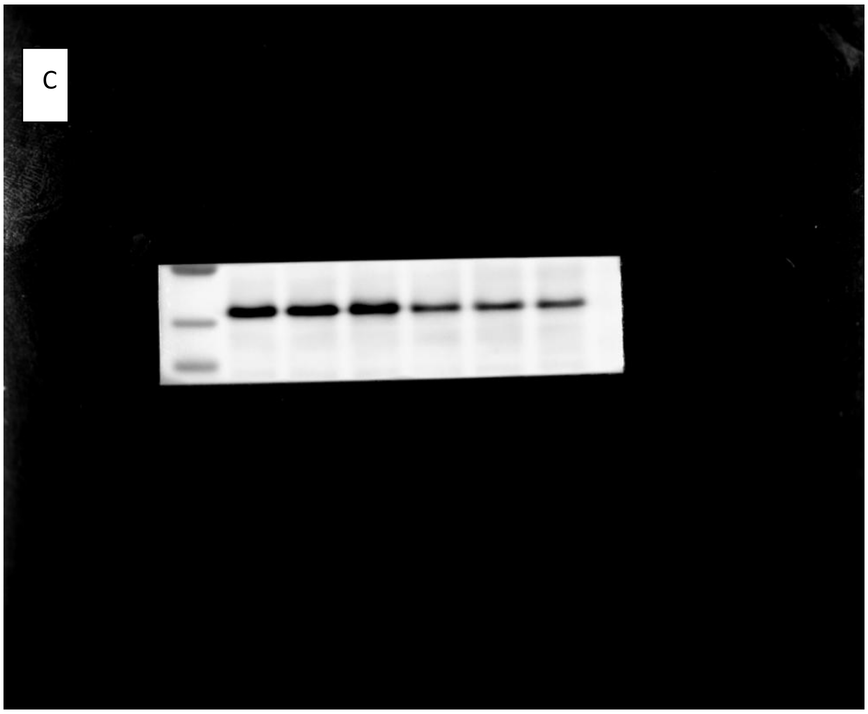

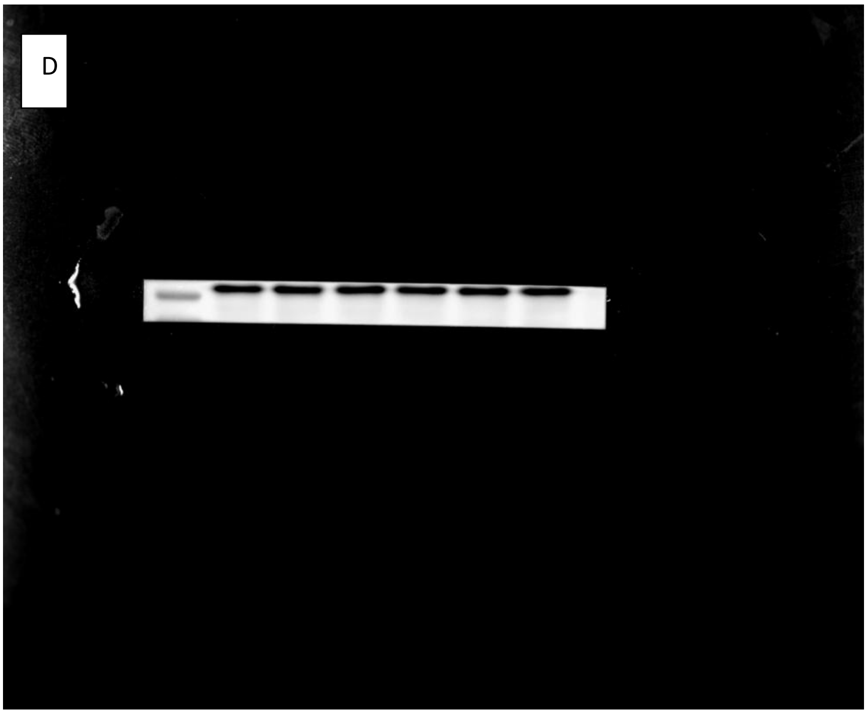

supplementary Figure3：

Figure 3A: Full-length, uncropped Western blot gel with molecular weight markers and all lanes.

Figure 3B: Cropped Western blot gel highlighting regions of interest.

Figure 3C: Western blot detection of TSHB protein following interference.

Figure 3D: Western blot detection of GAPDH as a housekeeping protein.

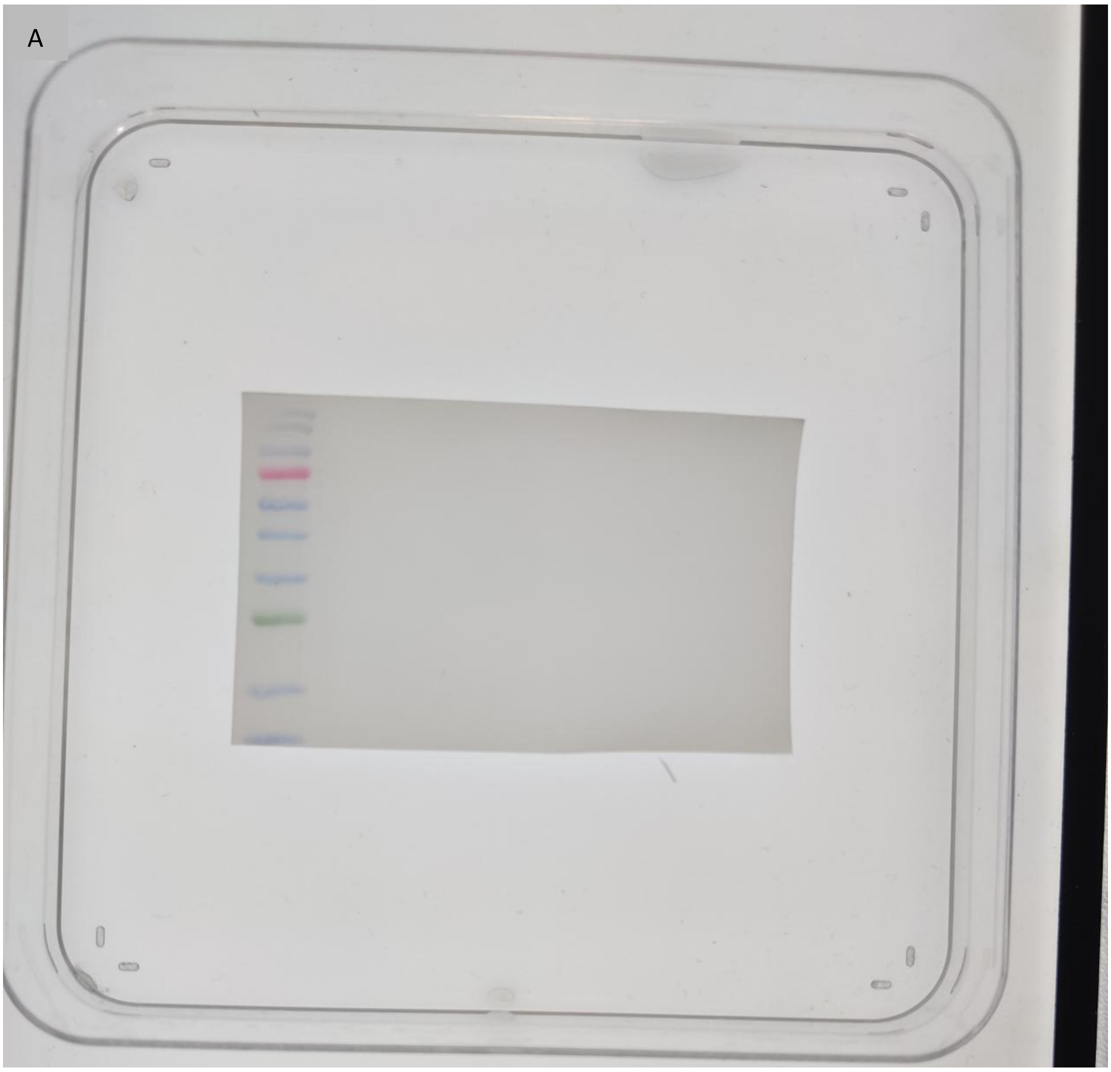
A

B

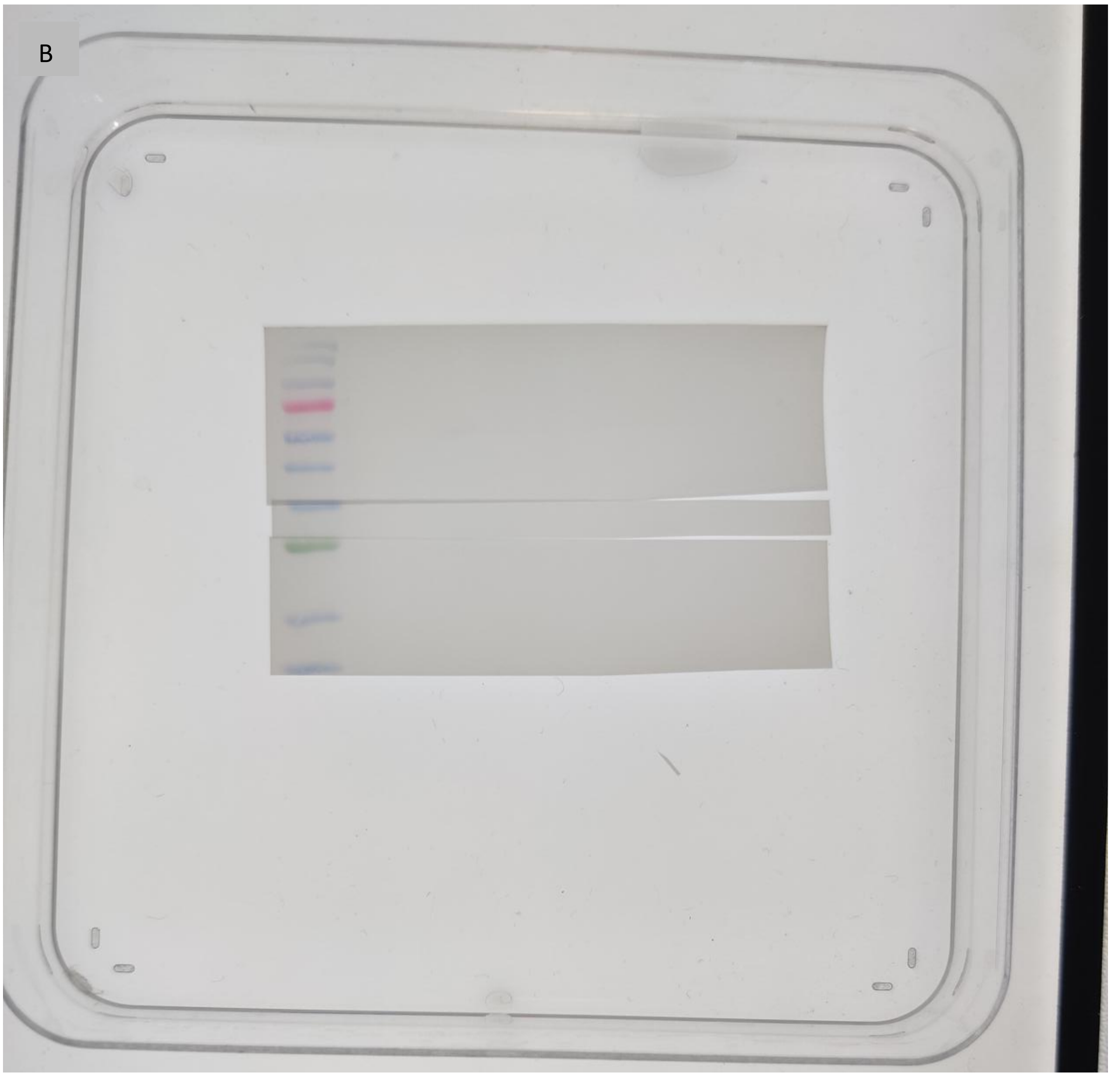

C

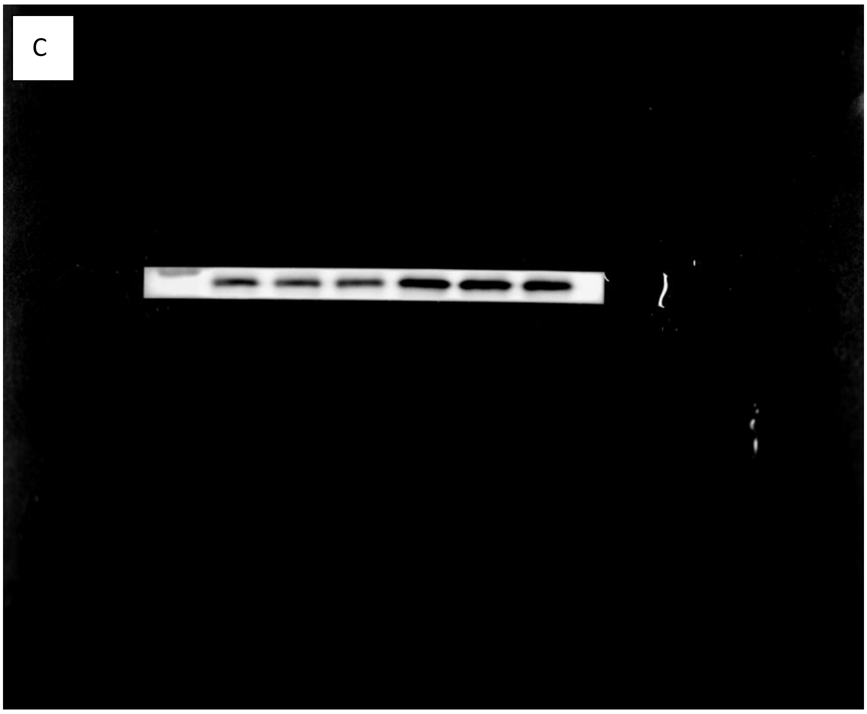

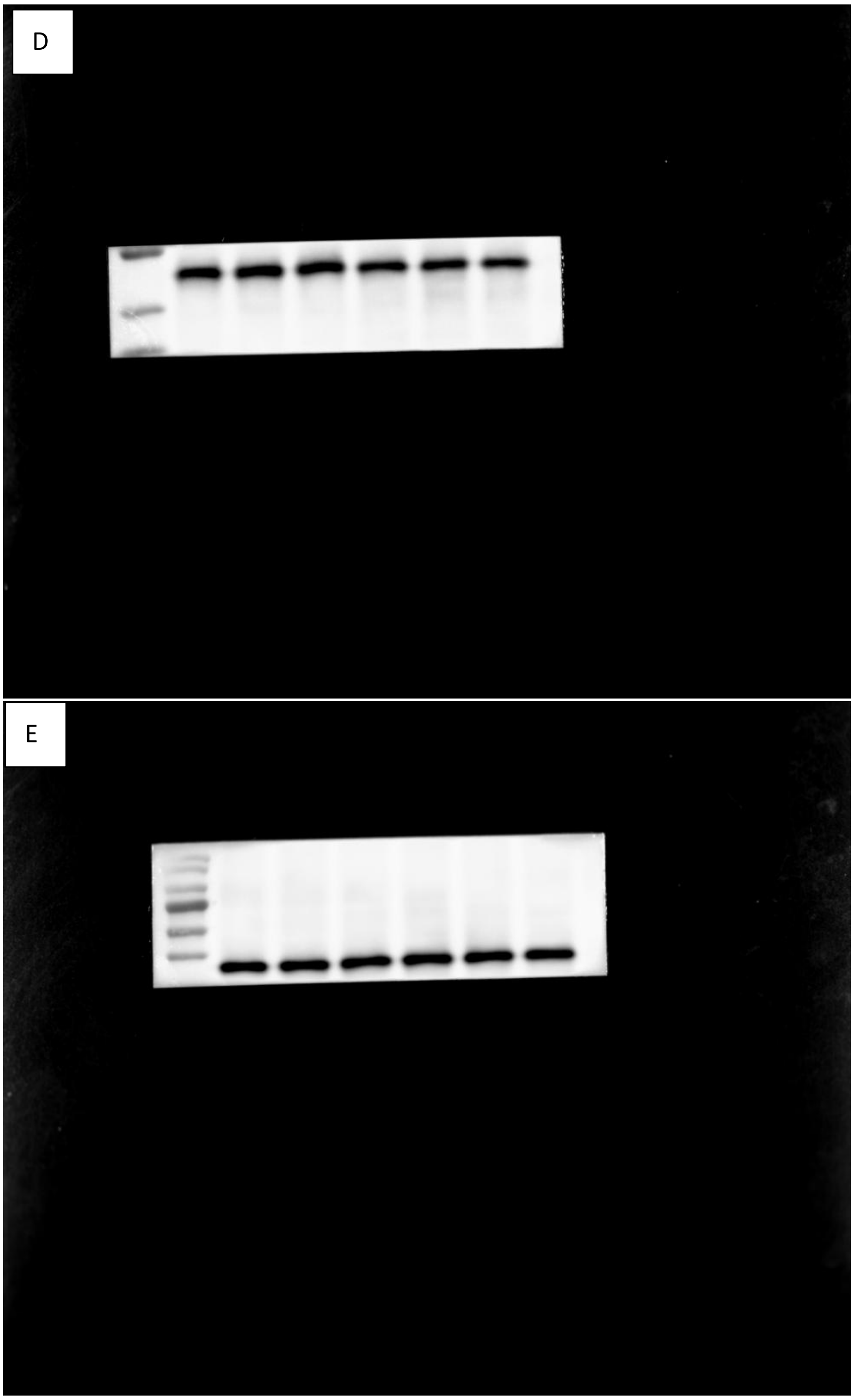

supplementary Figure4：

Figure 4A: Full-length, uncropped Western blot gel with molecular weight markers and all lanes.

Figure 4B: Cropped Western blot gel highlighting regions of interest.

Figure 4C: Western blot detection of CDK2 protein following overexpression.

Figure 4D: Western blot detection of BAX protein following overexpression.

Figure 4E: Western blot detection of β-action as a housekeeping protein.

A

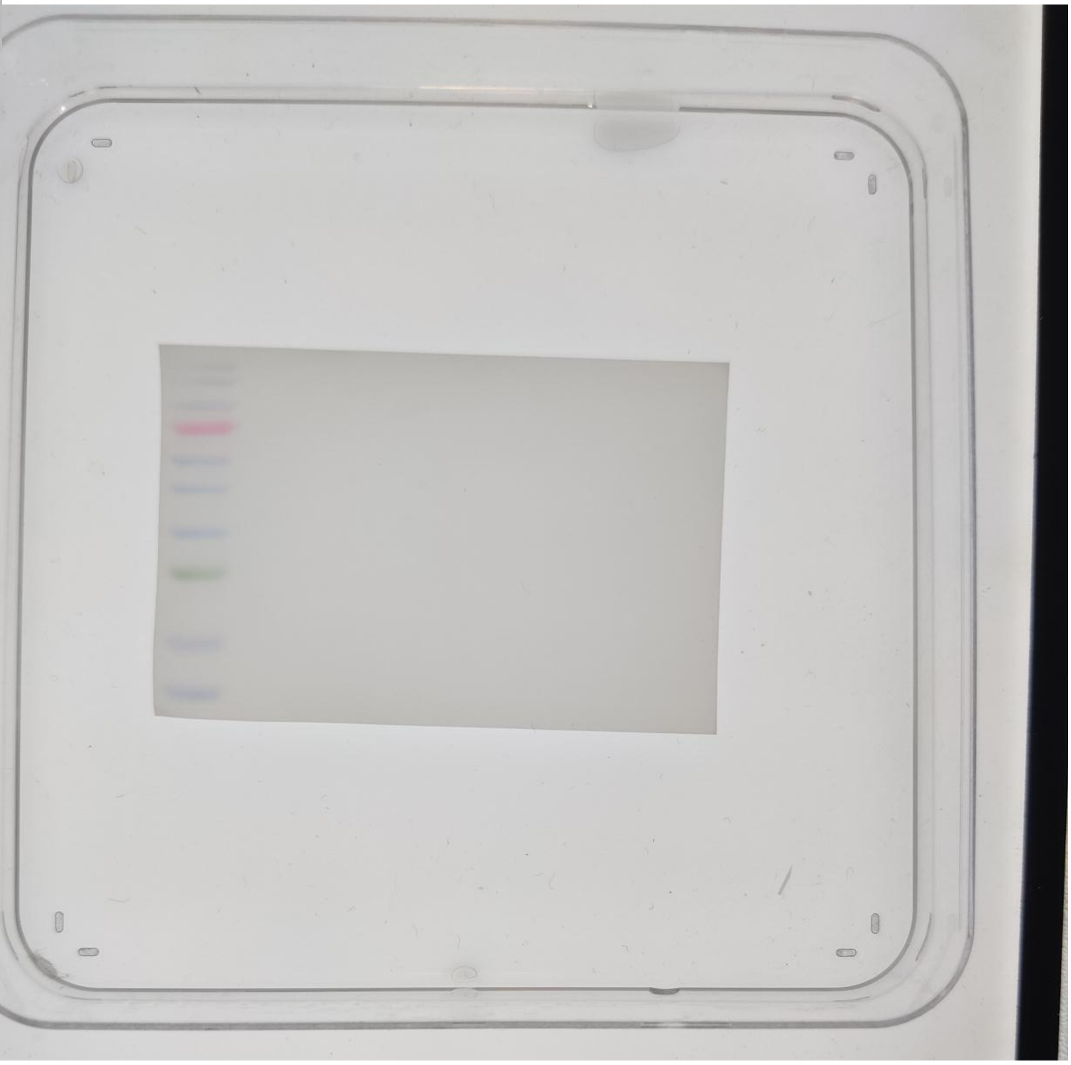

B

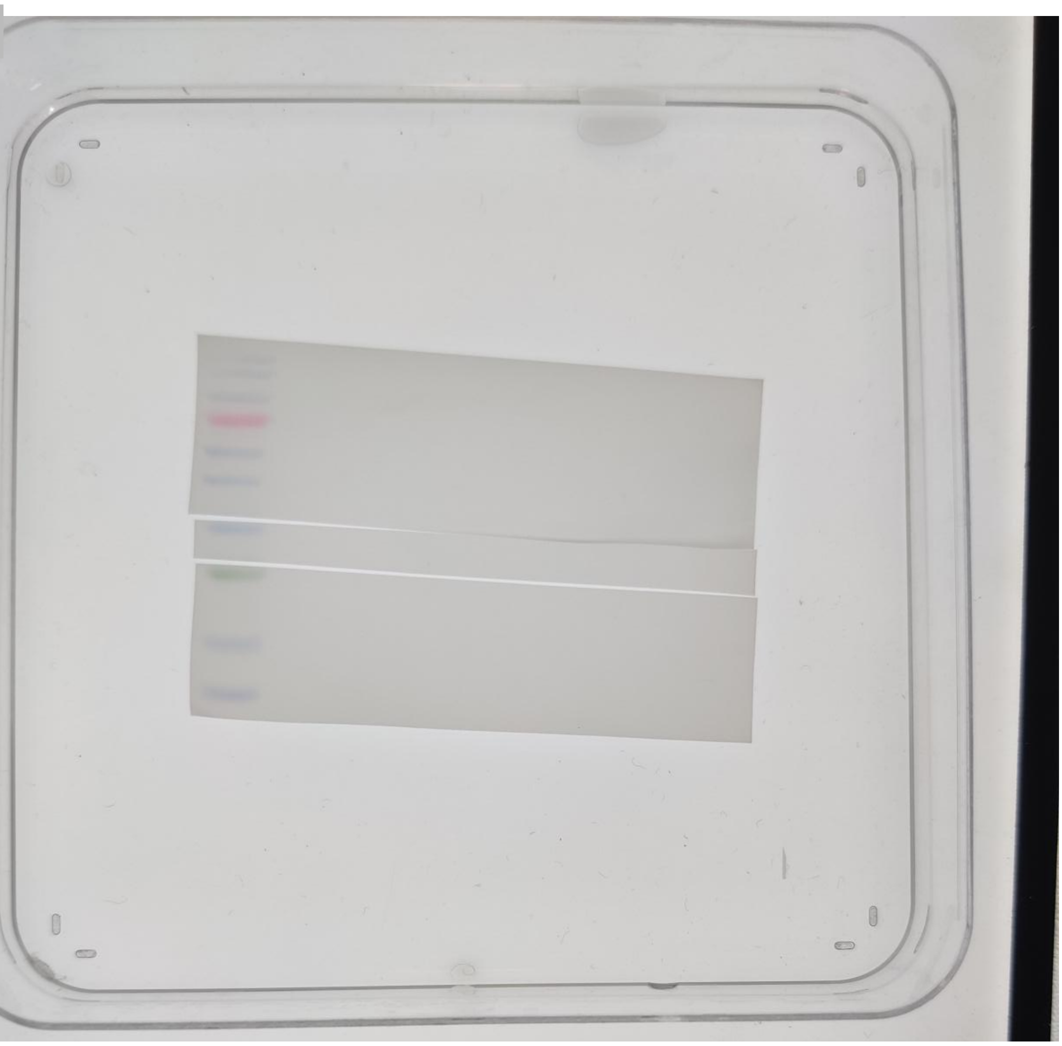

C

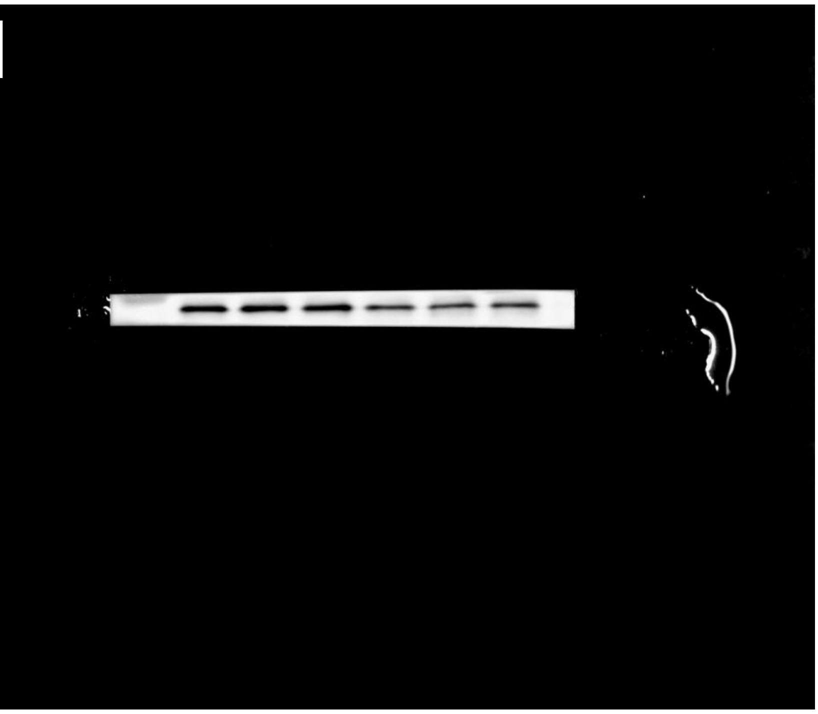

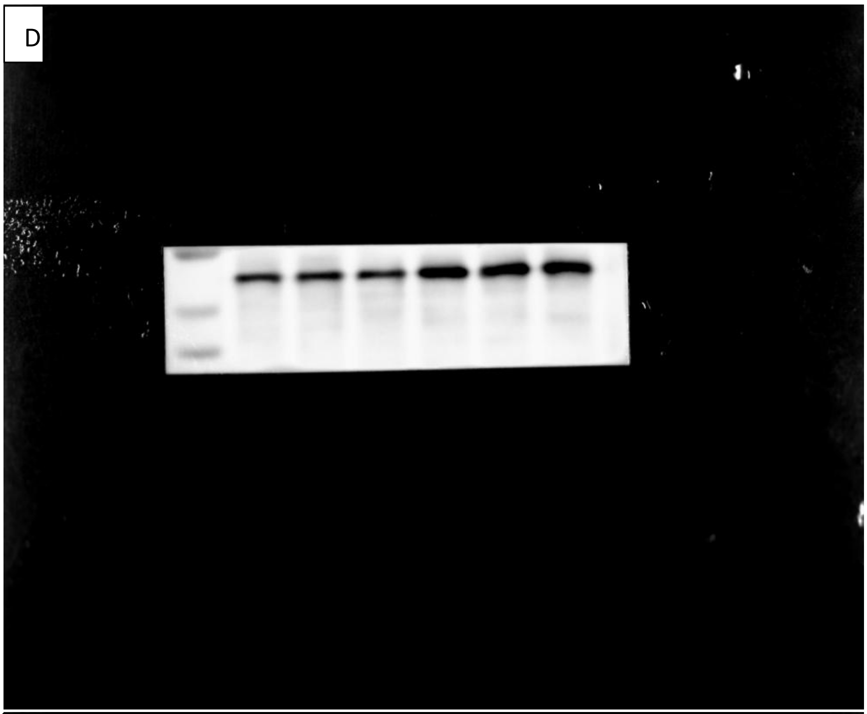

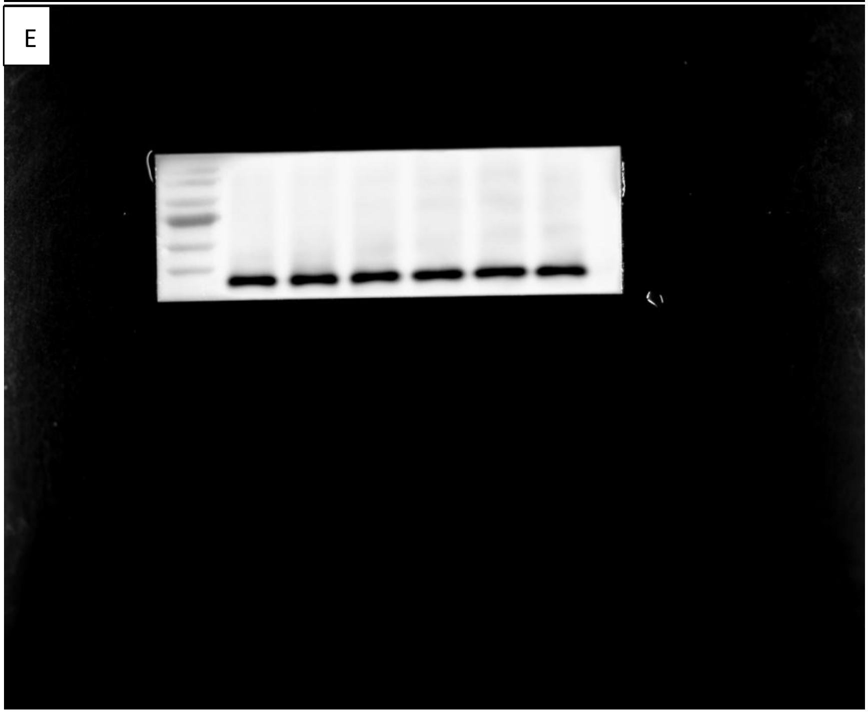

supplementary Figure5:

Figure 5A: Full-length, uncropped Western blot gel with molecular weight markers and all lanes.

Figure 5B: Cropped Western blot gel highlighting regions of interest.

Figure 5C: Western blot detection of CDK2 protein following interference.

Figure 5D: Western blot detection of BAX protein following interference.

Figure 4E: Western blot detection of β-action as a housekeeping protein.

A

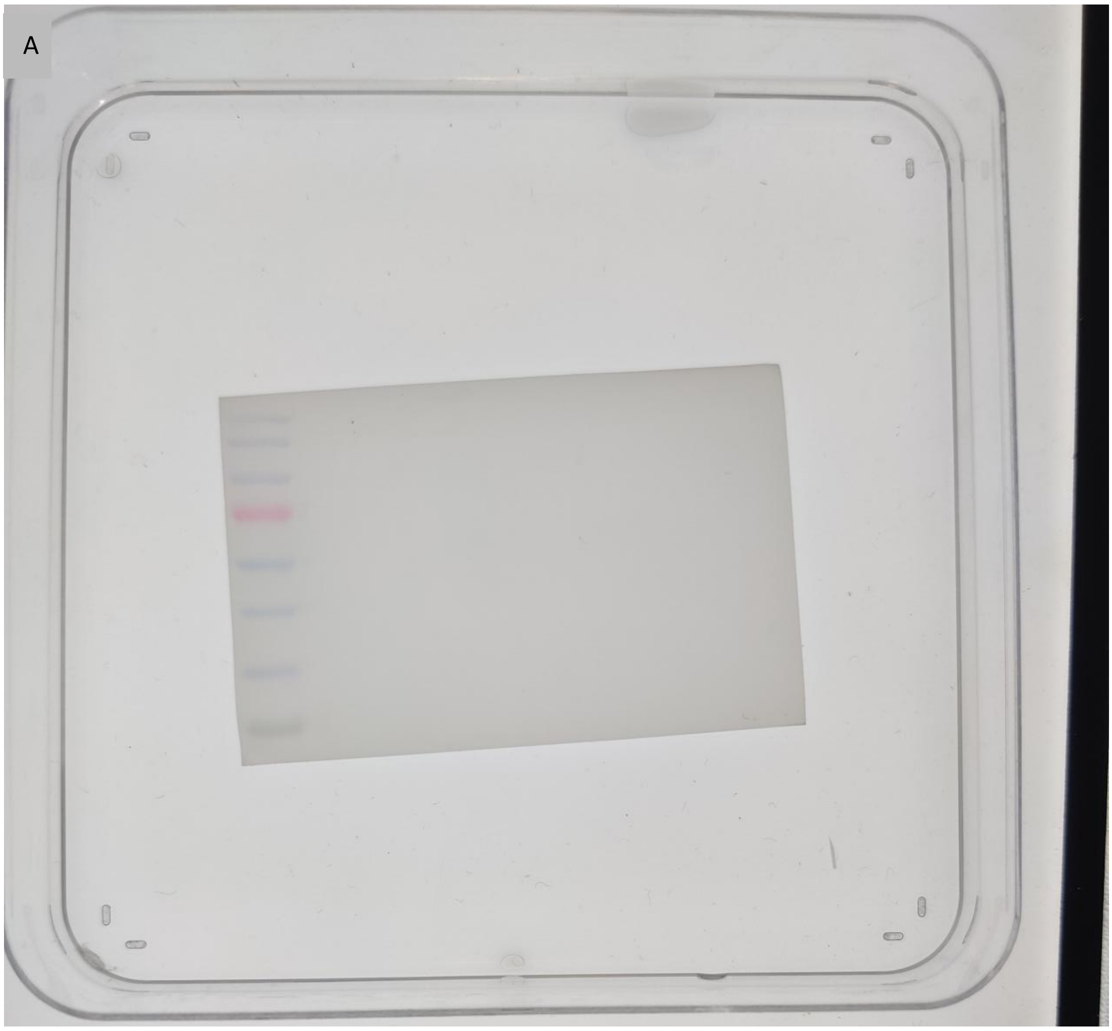

B

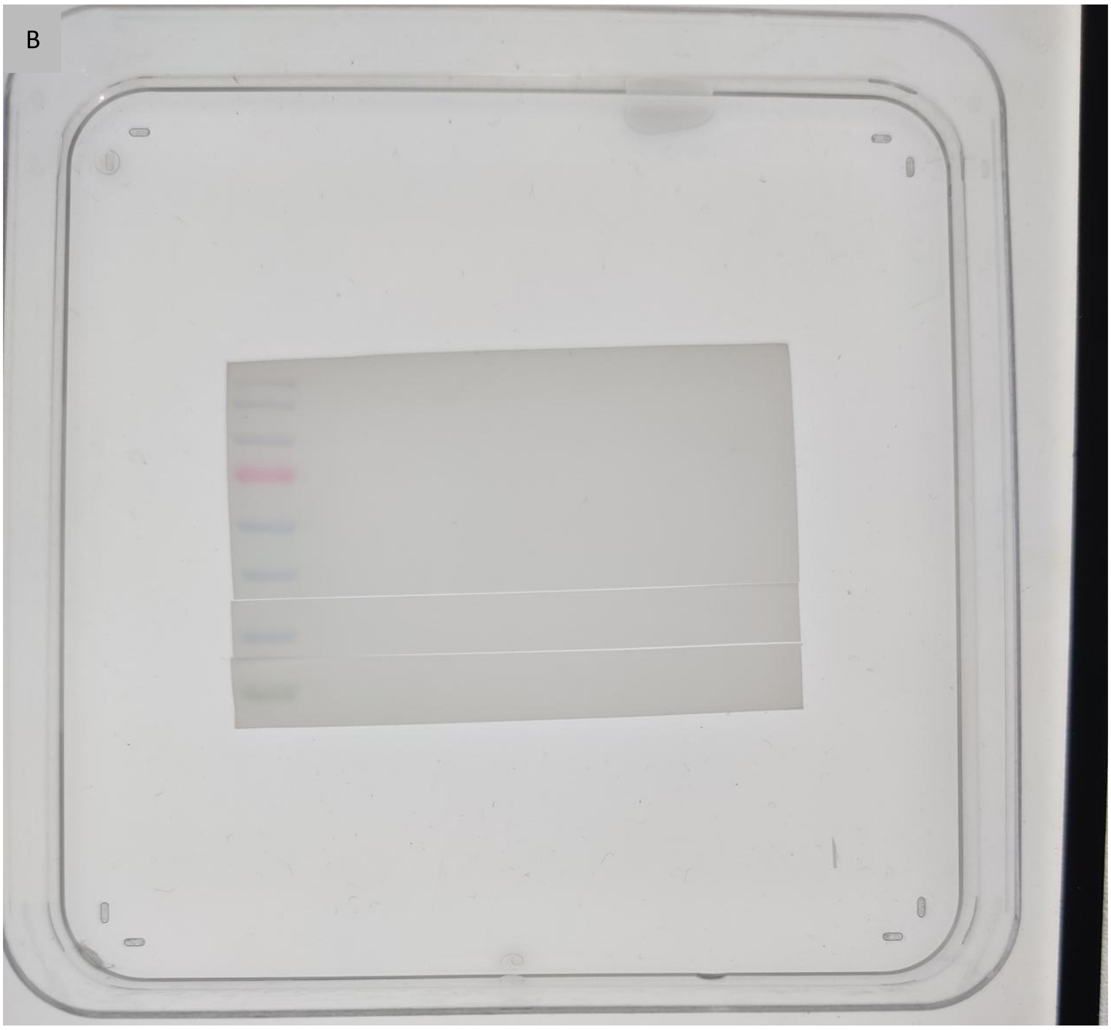

C

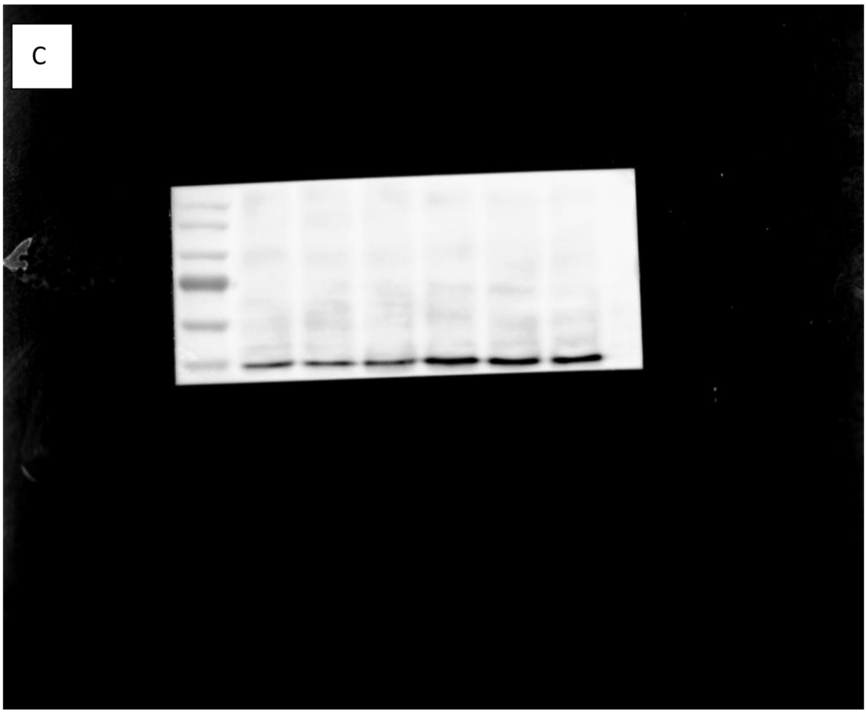

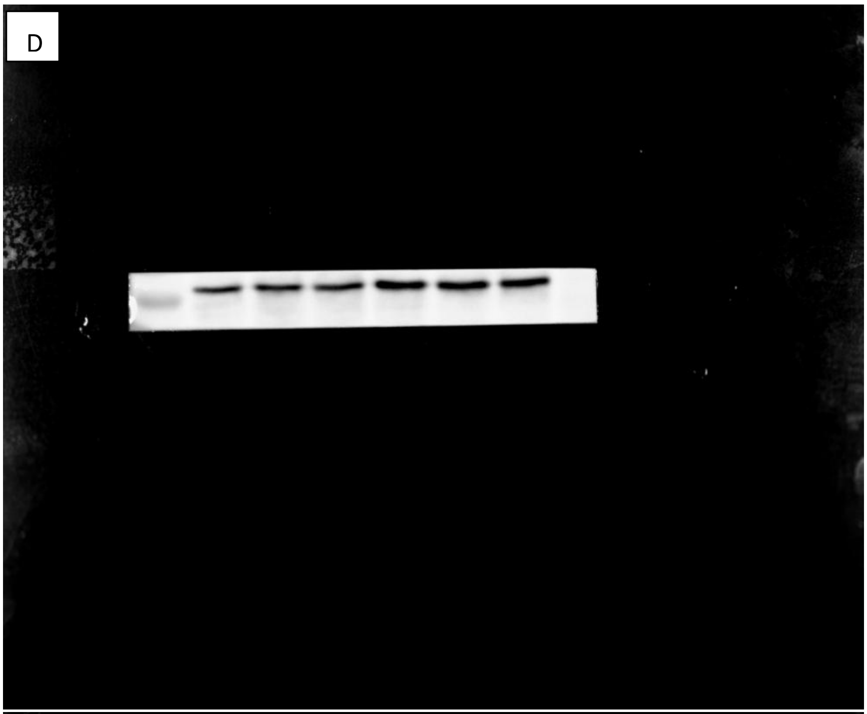

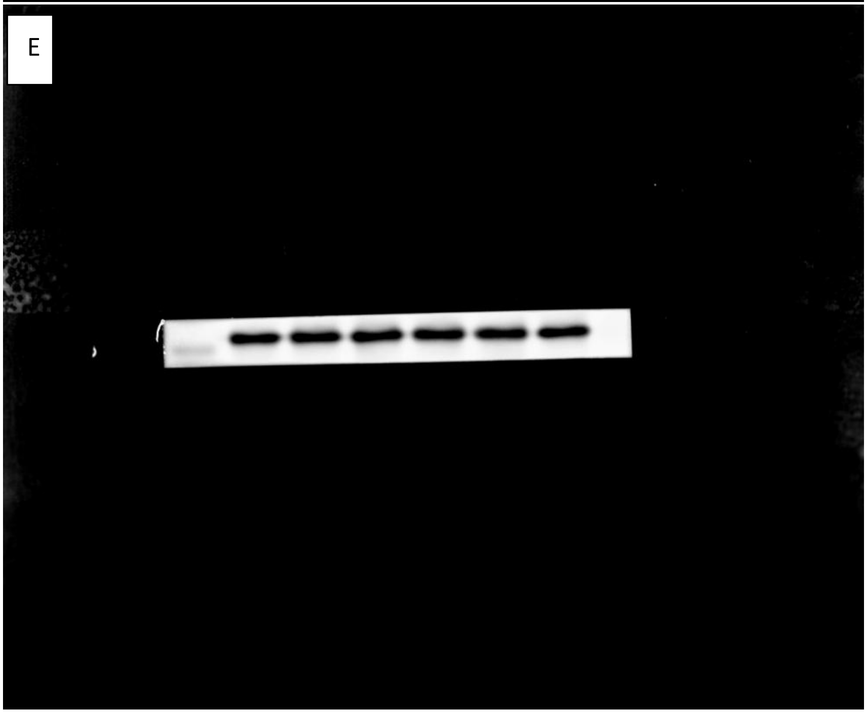

supplementary Figure6:

Figure 6A: Full-length, uncropped Western blot gel with molecular weight markers and all lanes.

Figure 6B: Cropped Western blot gel highlighting regions of interest.

Figure 6C: Western blot detection of CyclinE protein following overexpression.

Figure 6D: Western blot detection of Bcl-2 protein following overexpression.

Figure 6E: Western blot detection of GAPDH as a housekeeping protein.

A

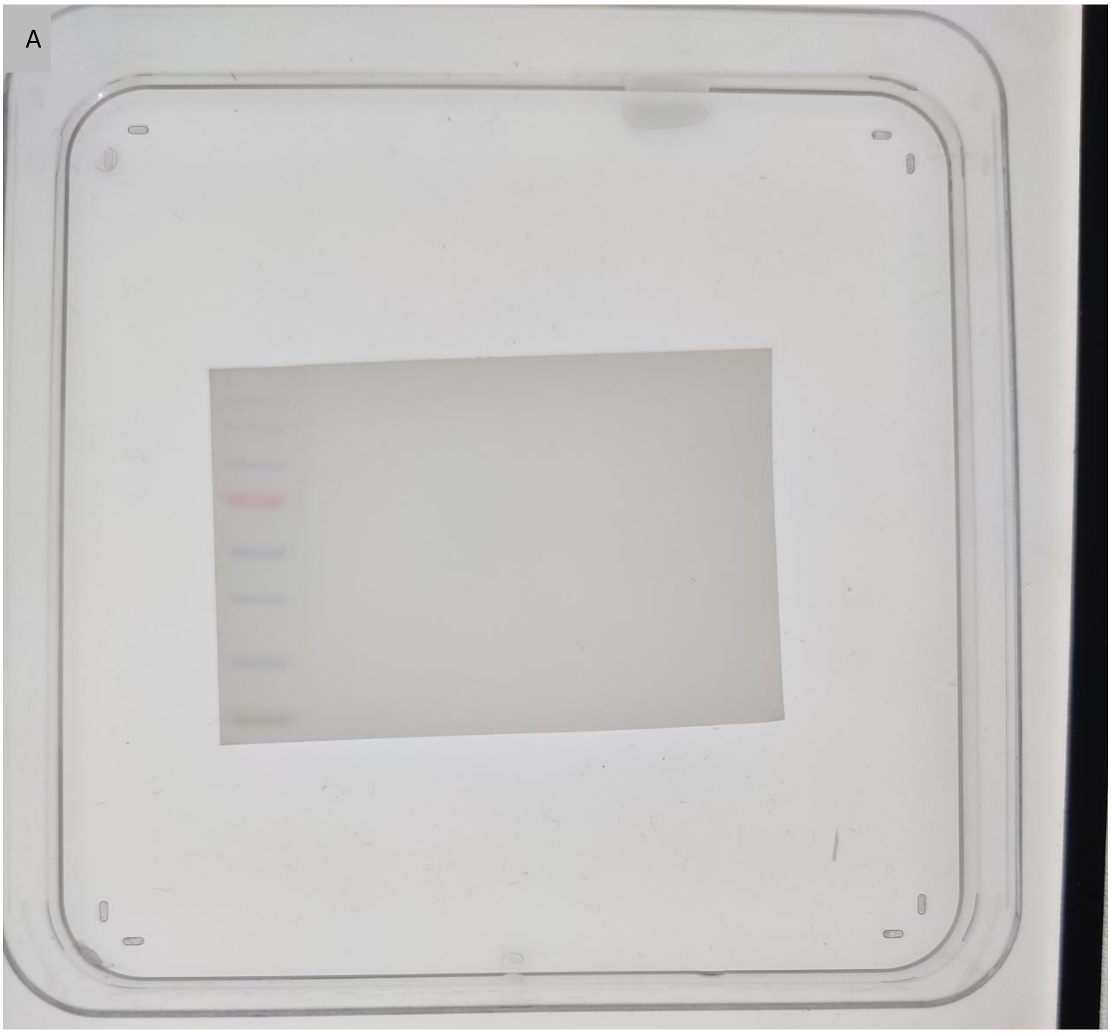

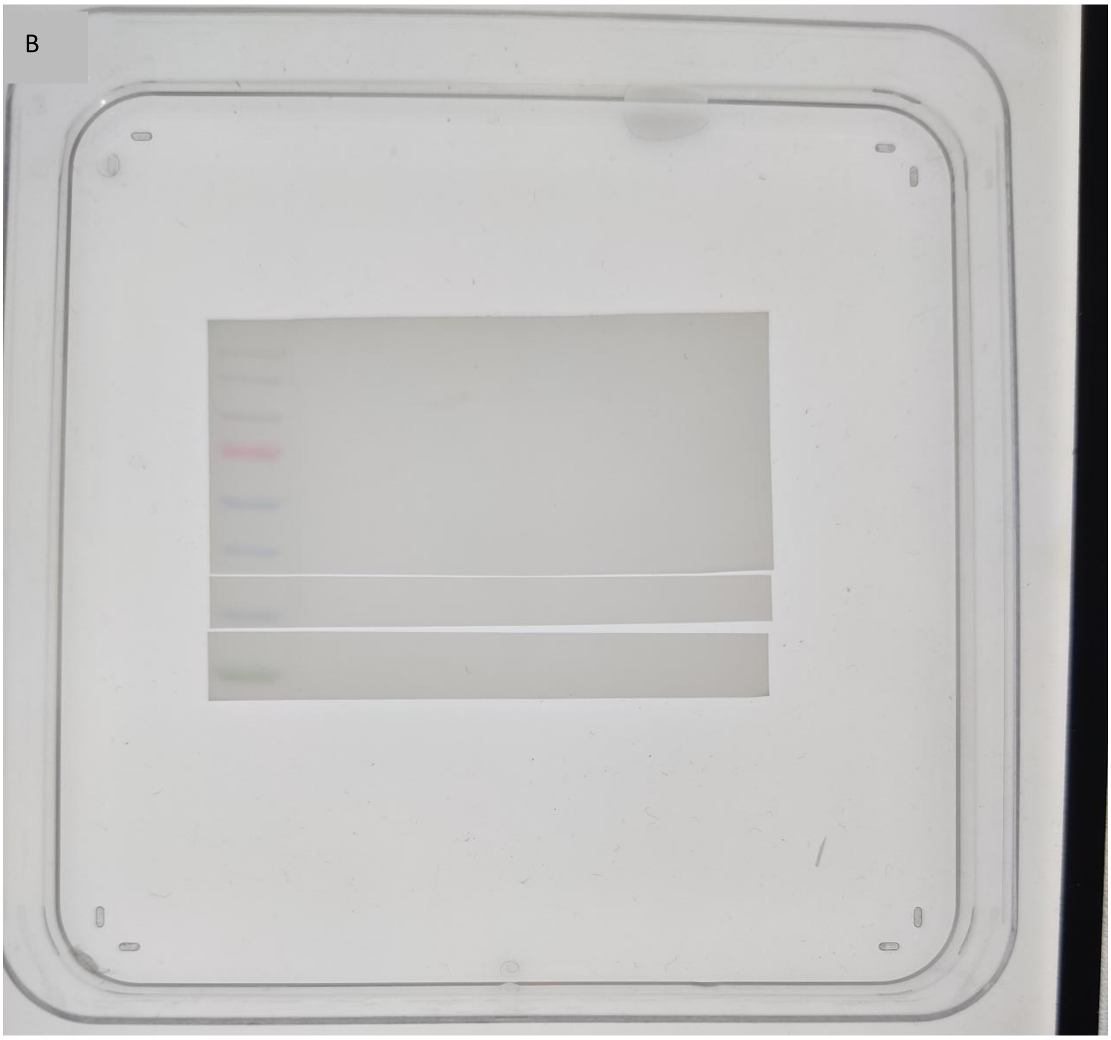
B

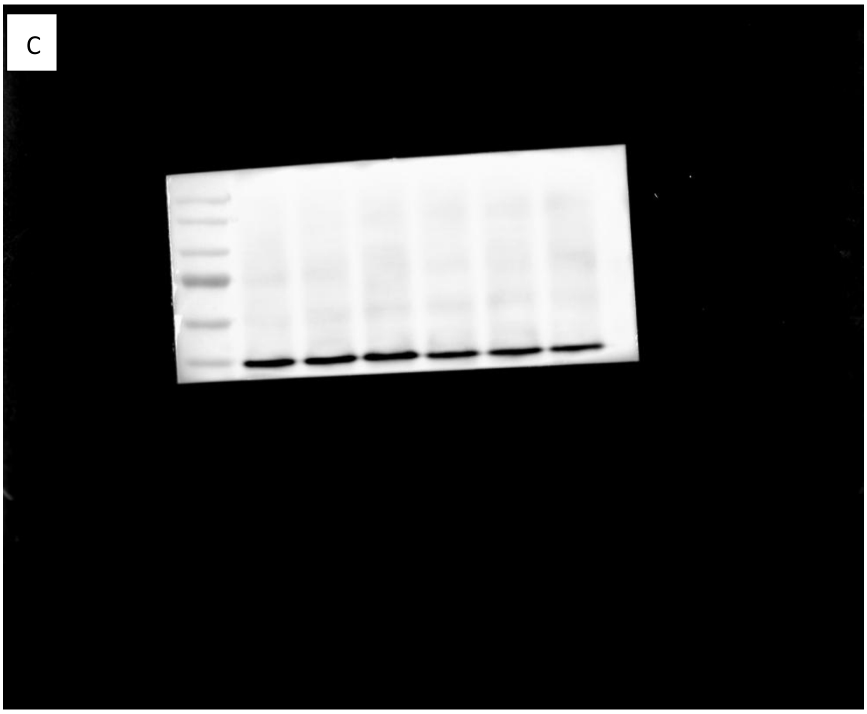
C

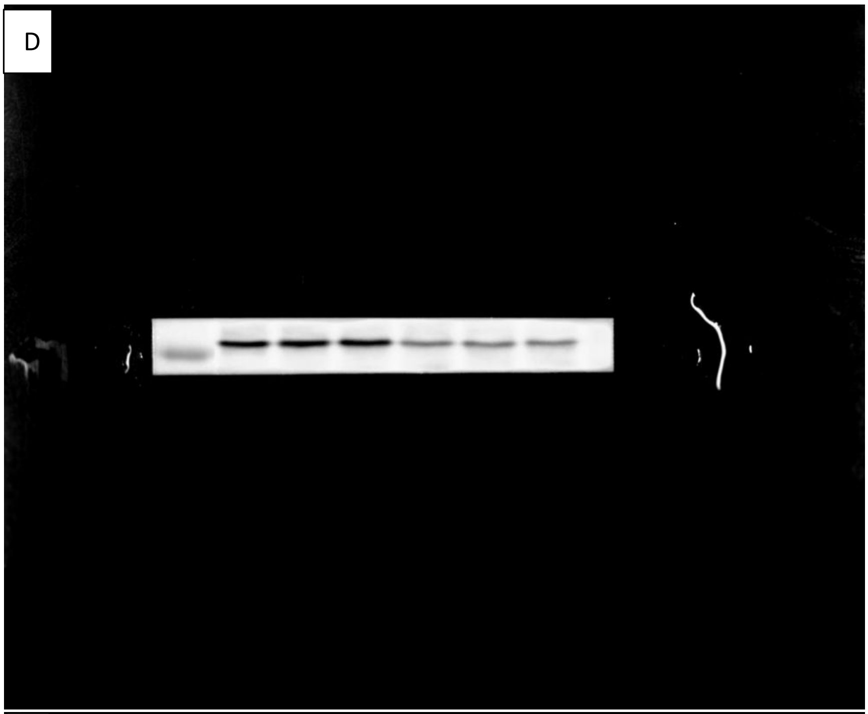

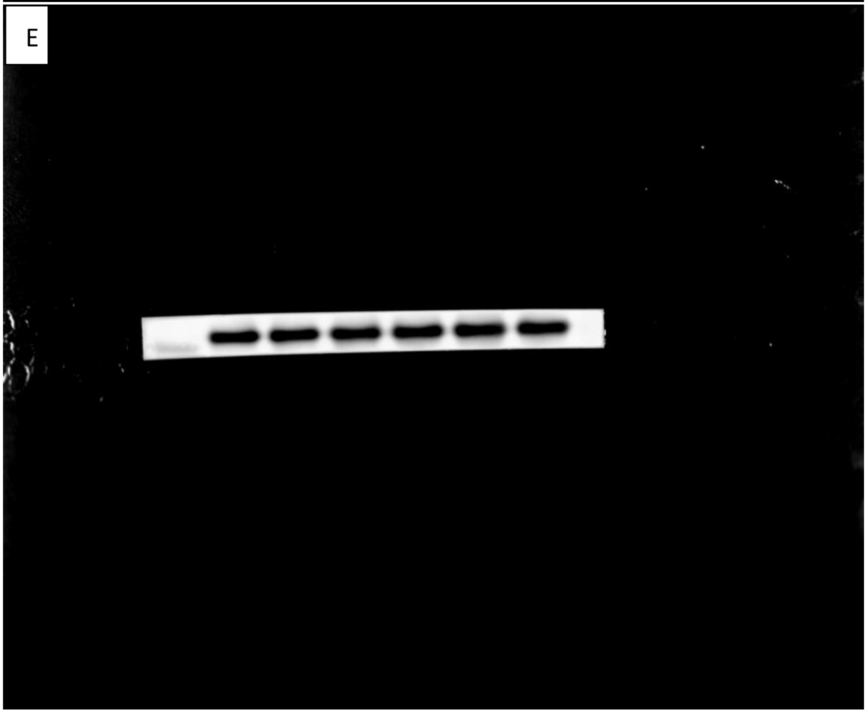

supplementary Figure7:

Figure 7A: Full-length, uncropped Western blot gel with molecular weight markers and all lanes.

Figure 7B: Cropped Western blot gel highlighting regions of interest.

Figure 7C: Western blot detection of CyclinE protein following interference.

Figure 7D: Western blot detection of Bcl-2 protein following interference.

Figure 7E: Western blot detection of GAPDH as a housekeeping protein.

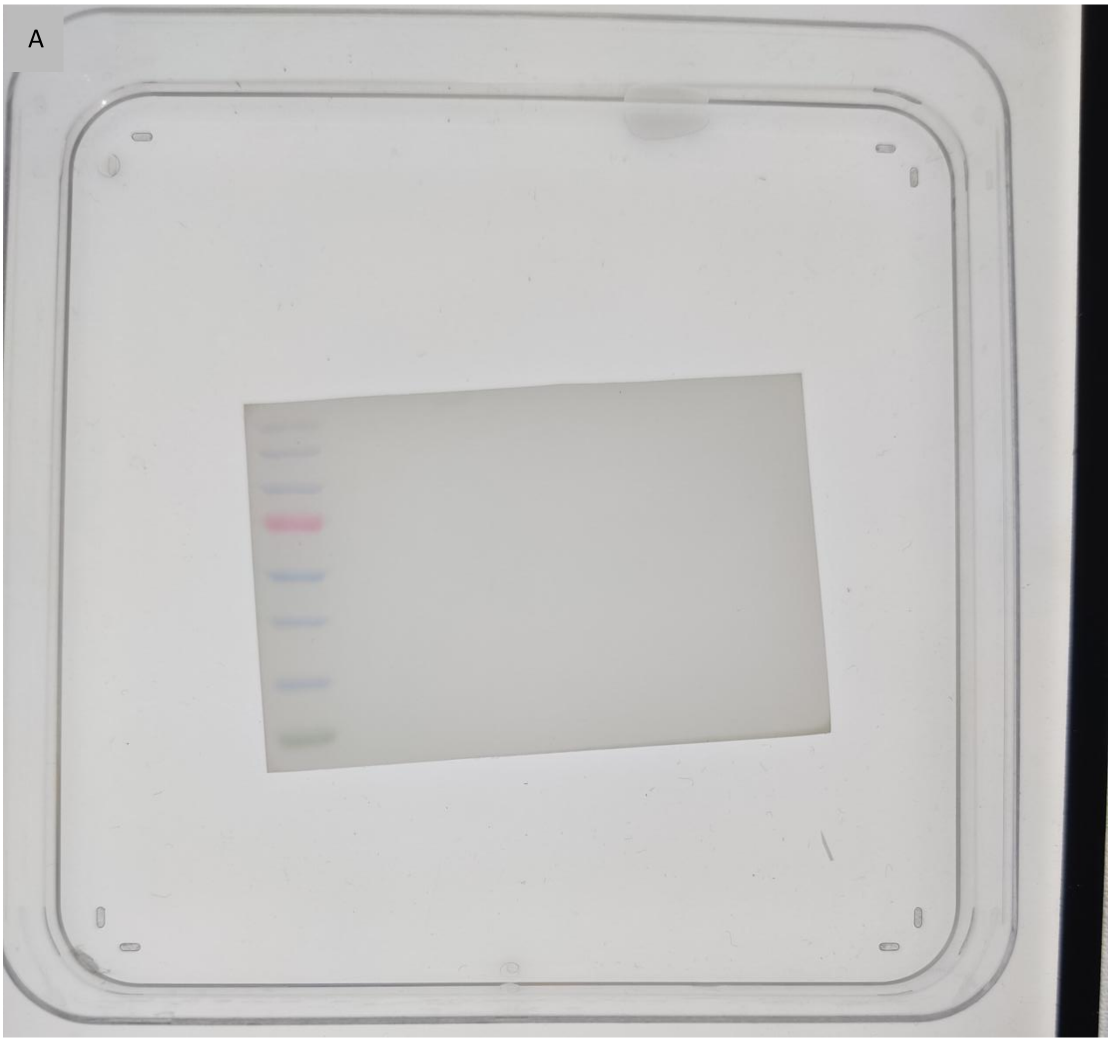
A

B

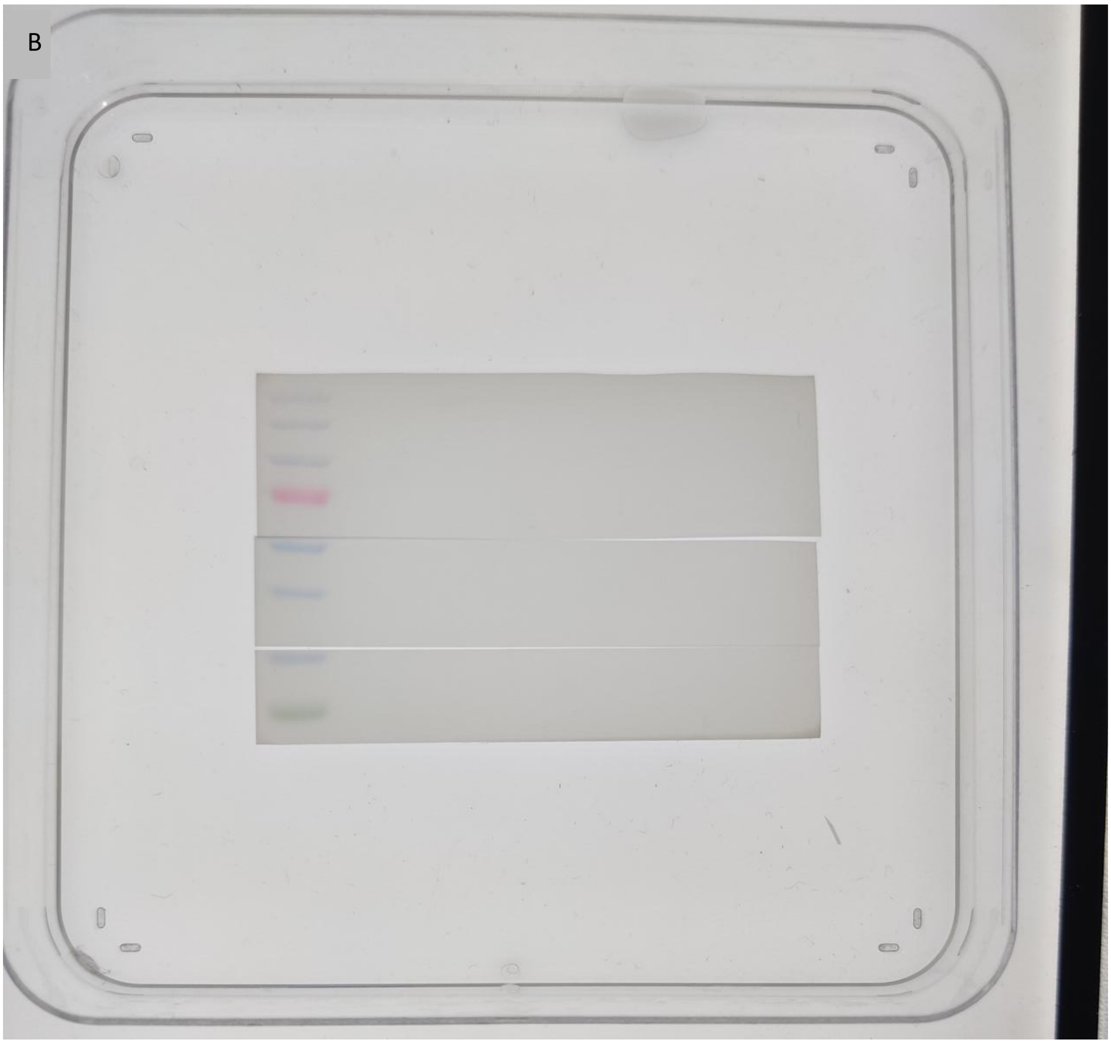

C

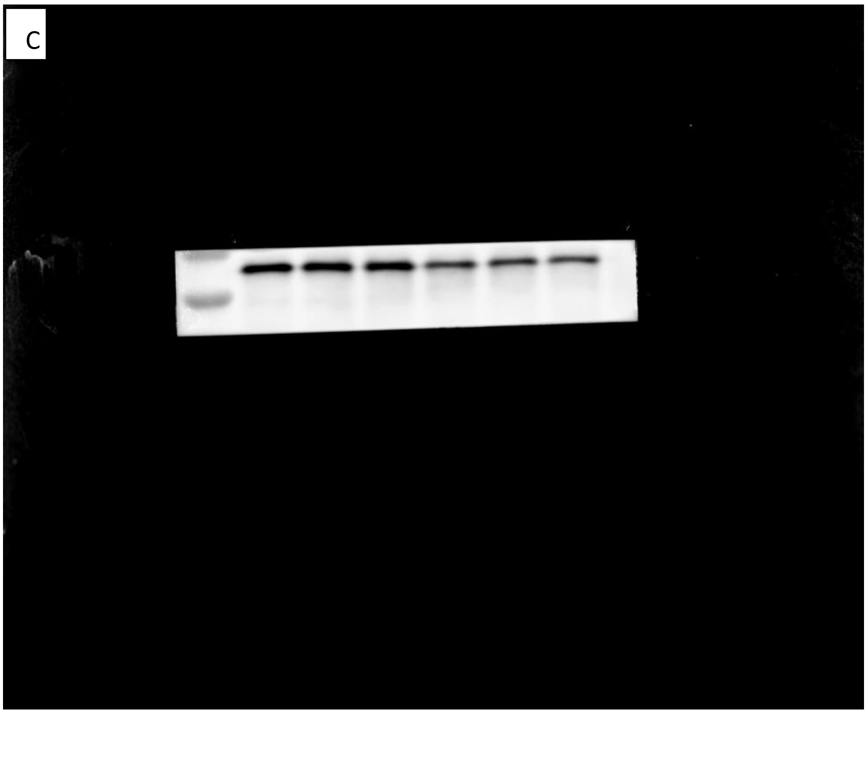

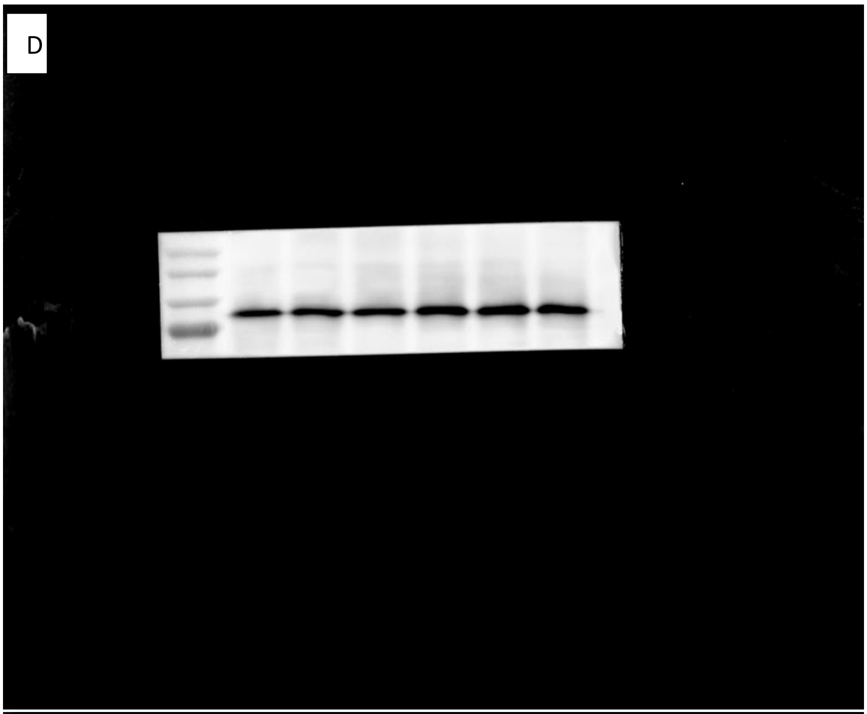

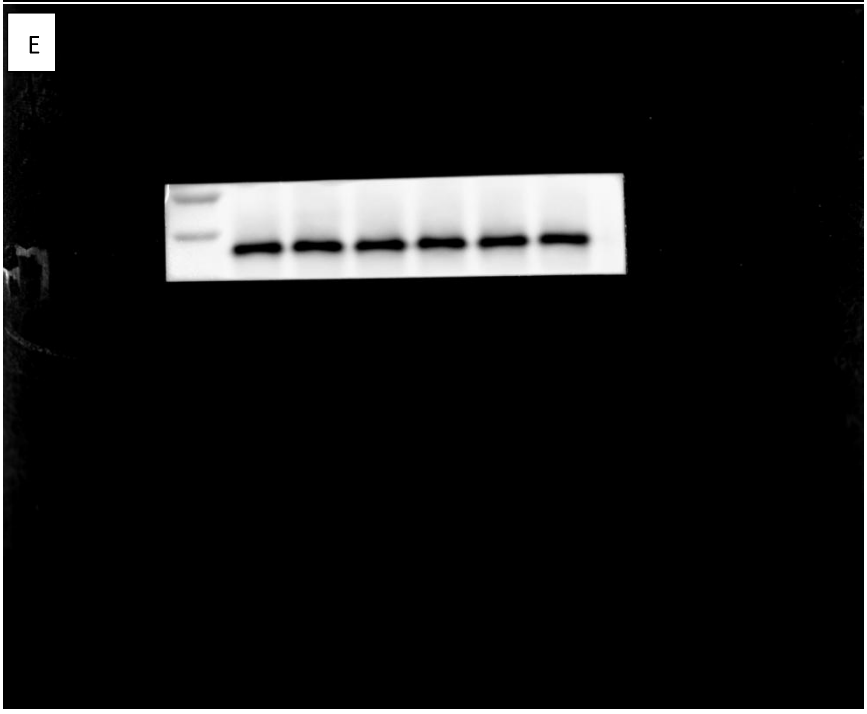

supplementary Figure8:

Due to the distinct focus of each protein (Caspase3 and TSHR) in separate sections of the article, the Western blot results for each protein are presented individually in the figures.

Figure 8A: Full-length, uncropped Western blot gel with molecular weight markers and all lanes.

Figure 8B: Cropped Western blot gel highlighting regions of interest.

Figure 8C: Western blot detection of Caspase3 protein following overexpression.

Figure 8D: Western blot detection of TSHR protein following overexpression.

Figure 8E: Western blot detection of β-action as a housekeeping protein.

A

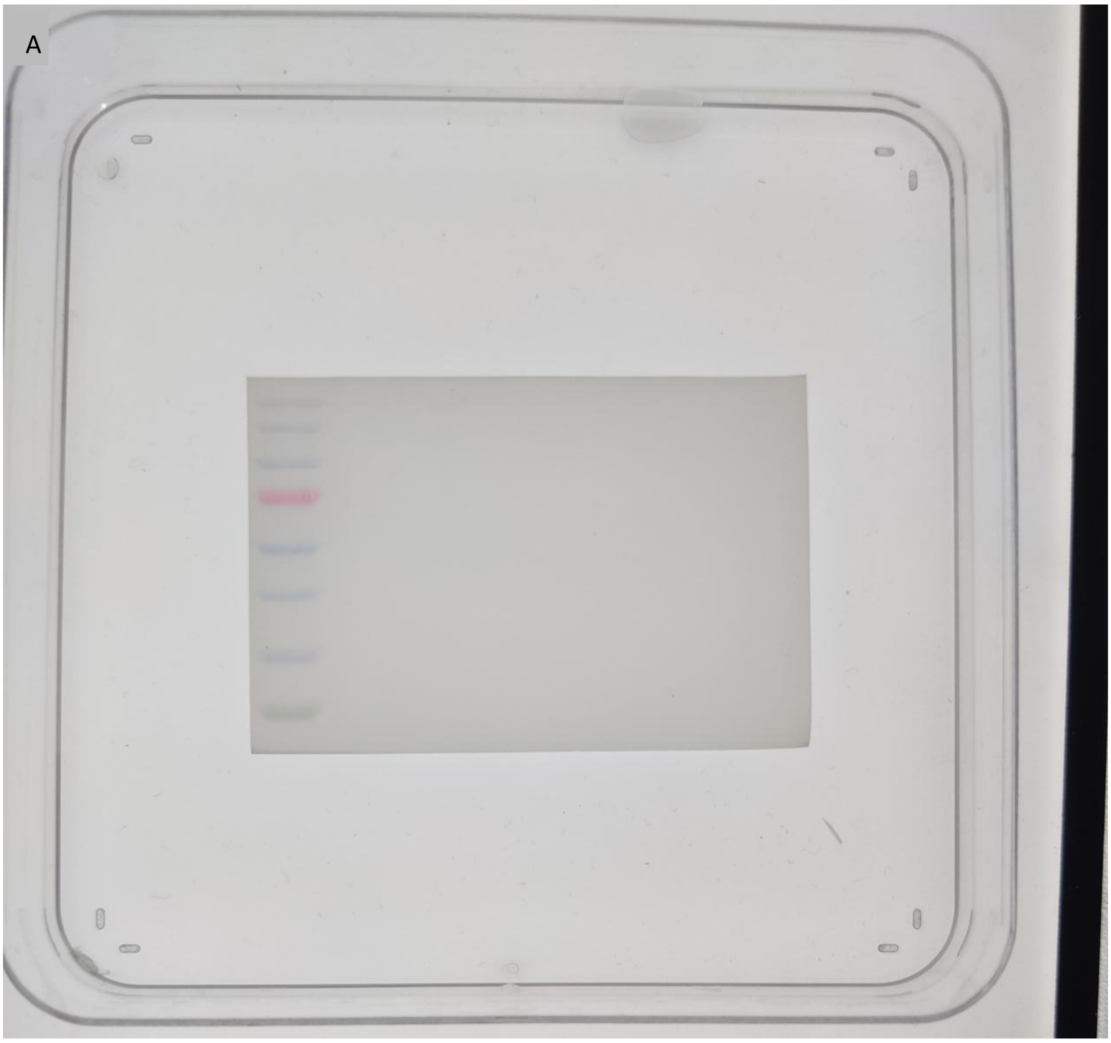

B

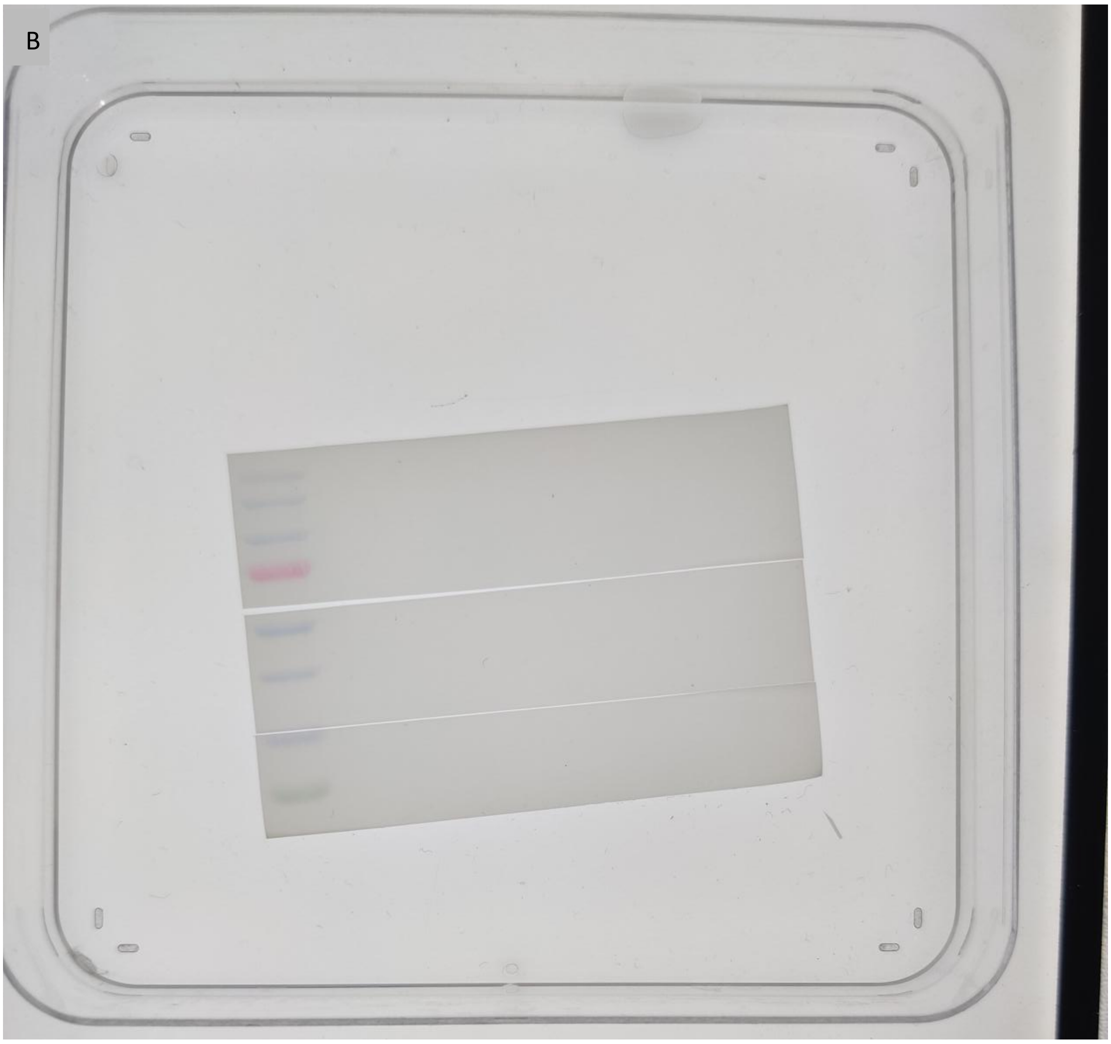

C

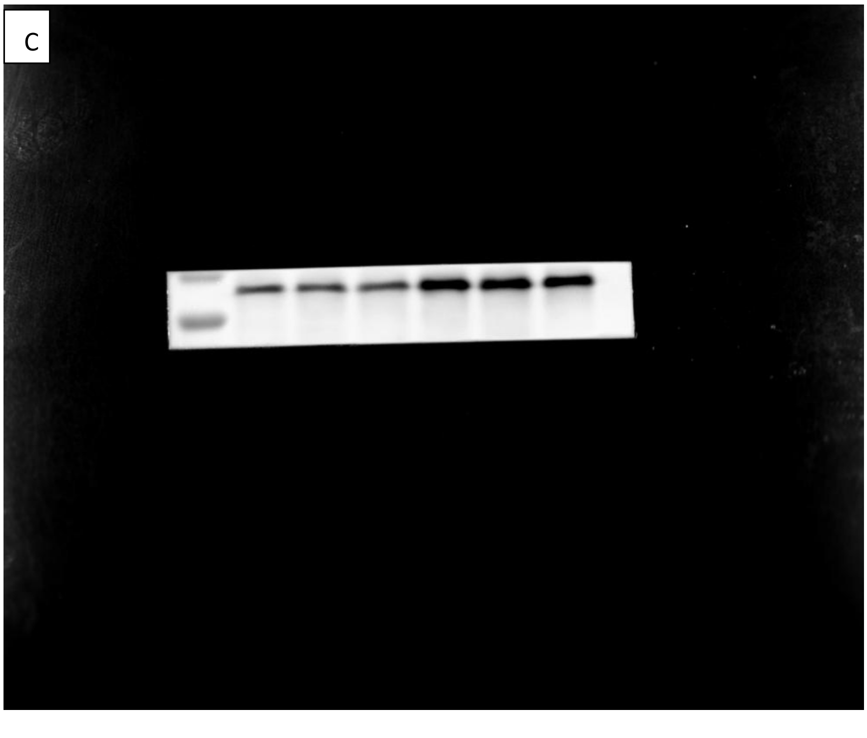

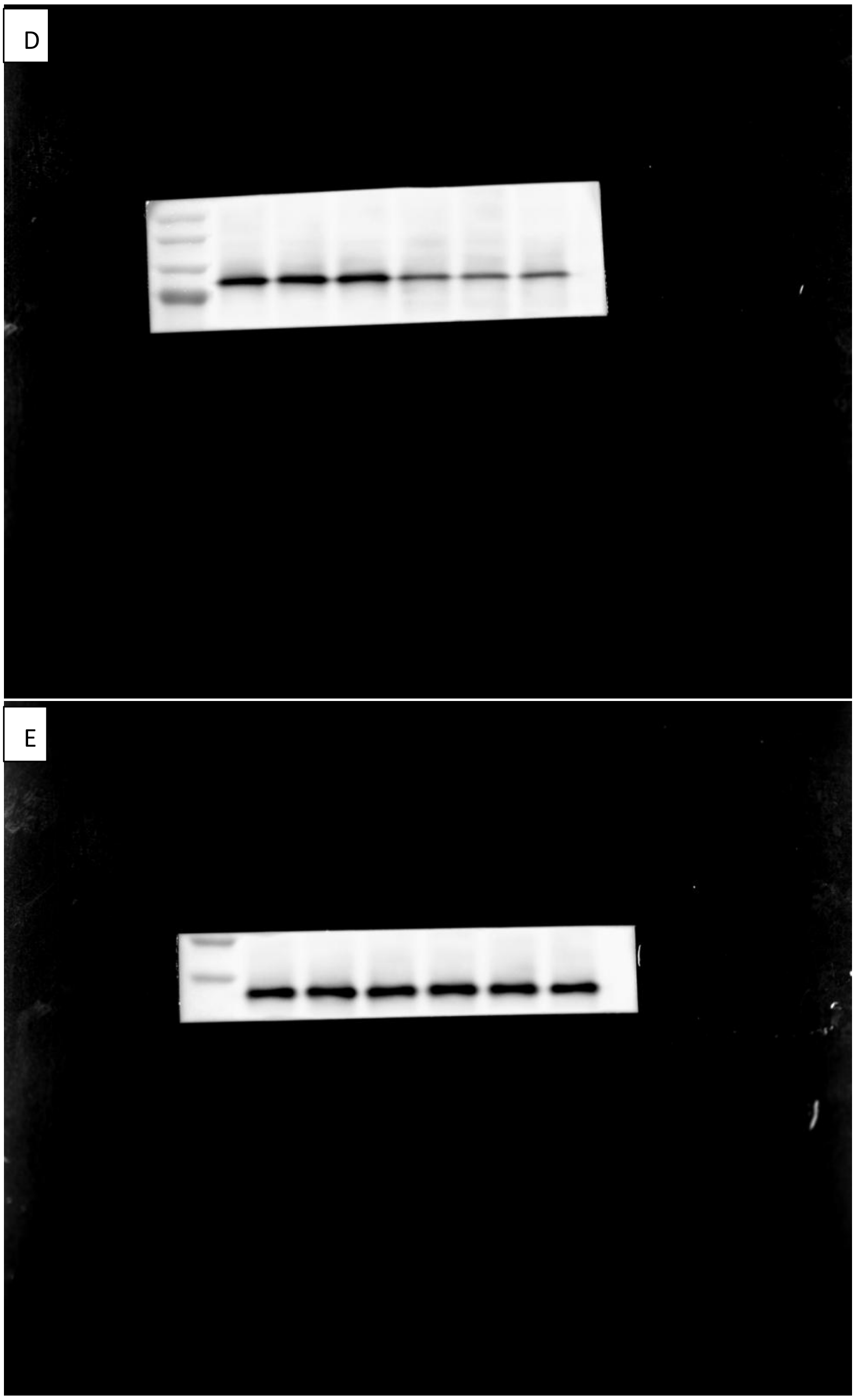

supplementary Figure9:

Due to the distinct focus of each protein (Caspase3 and TSHR) in separate sections of the article, the Western blot results for each protein are presented individually in the figures.

Figure 9A: Full-length, uncropped Western blot gel with molecular weight markers and all lanes.

Figure 9B: Cropped Western blot gel highlighting regions of interest.

Figure 9C: Western blot detection of Caspase3 protein following interference.

Figure 9D: Western blot detection of TSHR protein following interference.

Figure 9E: Western blot detection of β-action as a housekeeping protein.

A

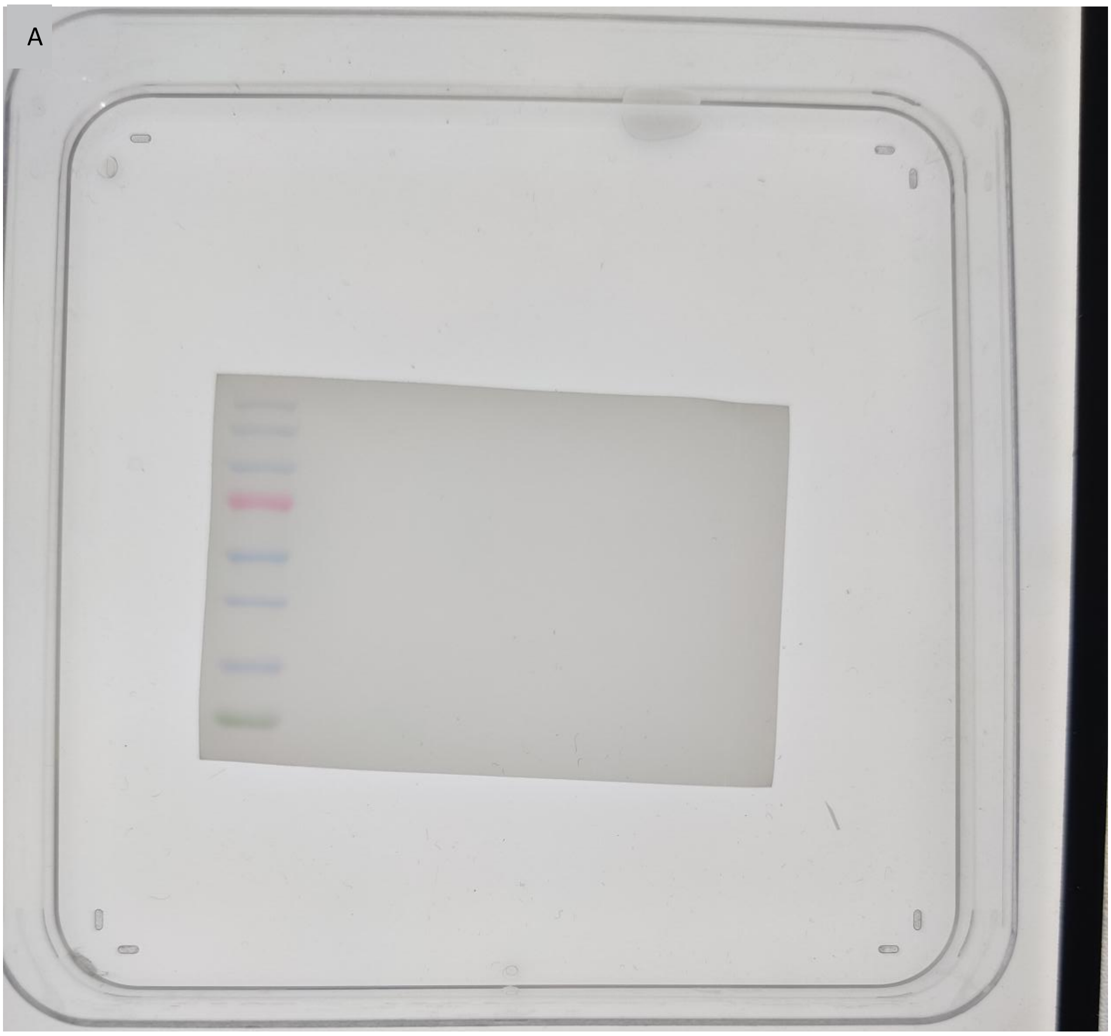

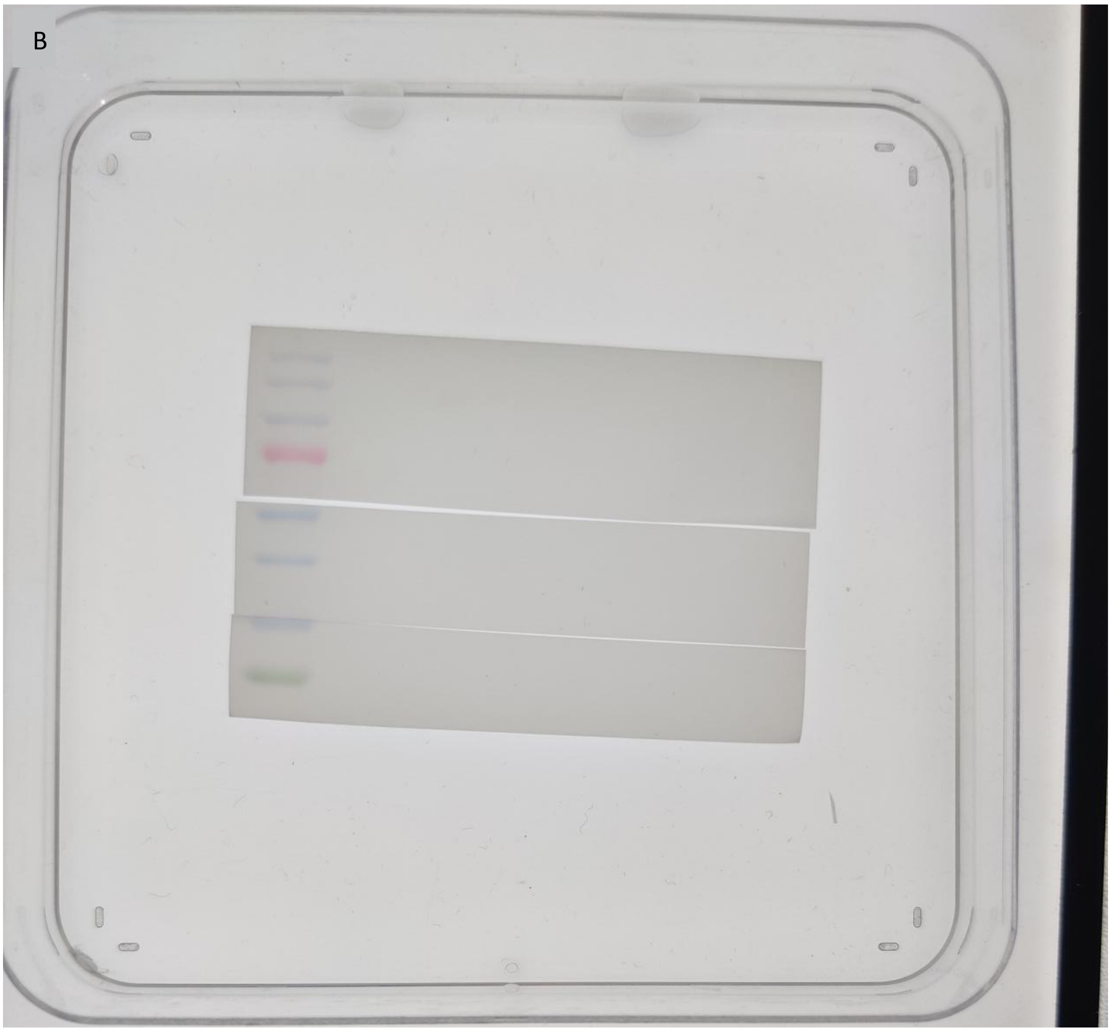
B

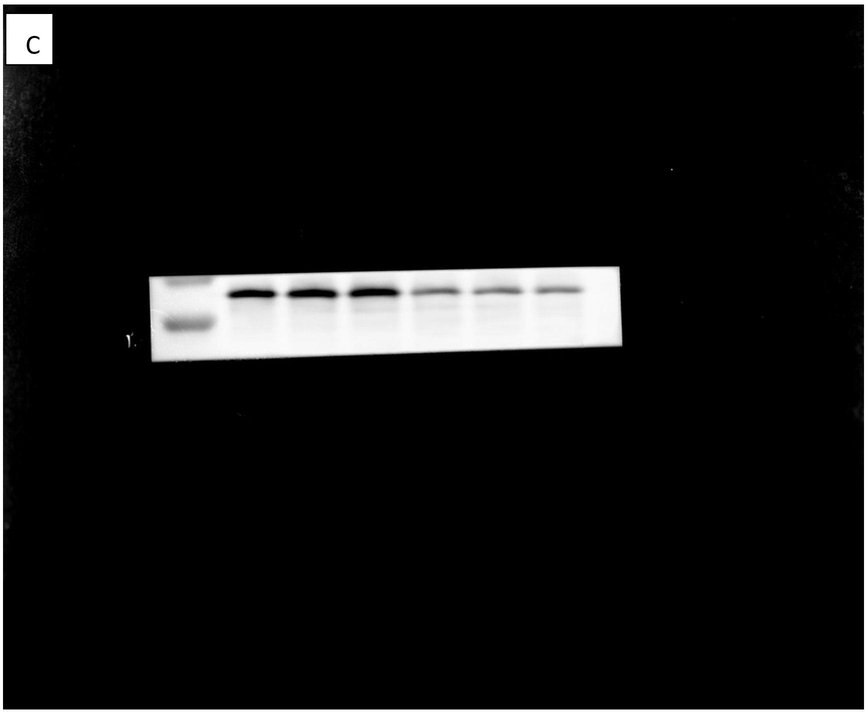
C

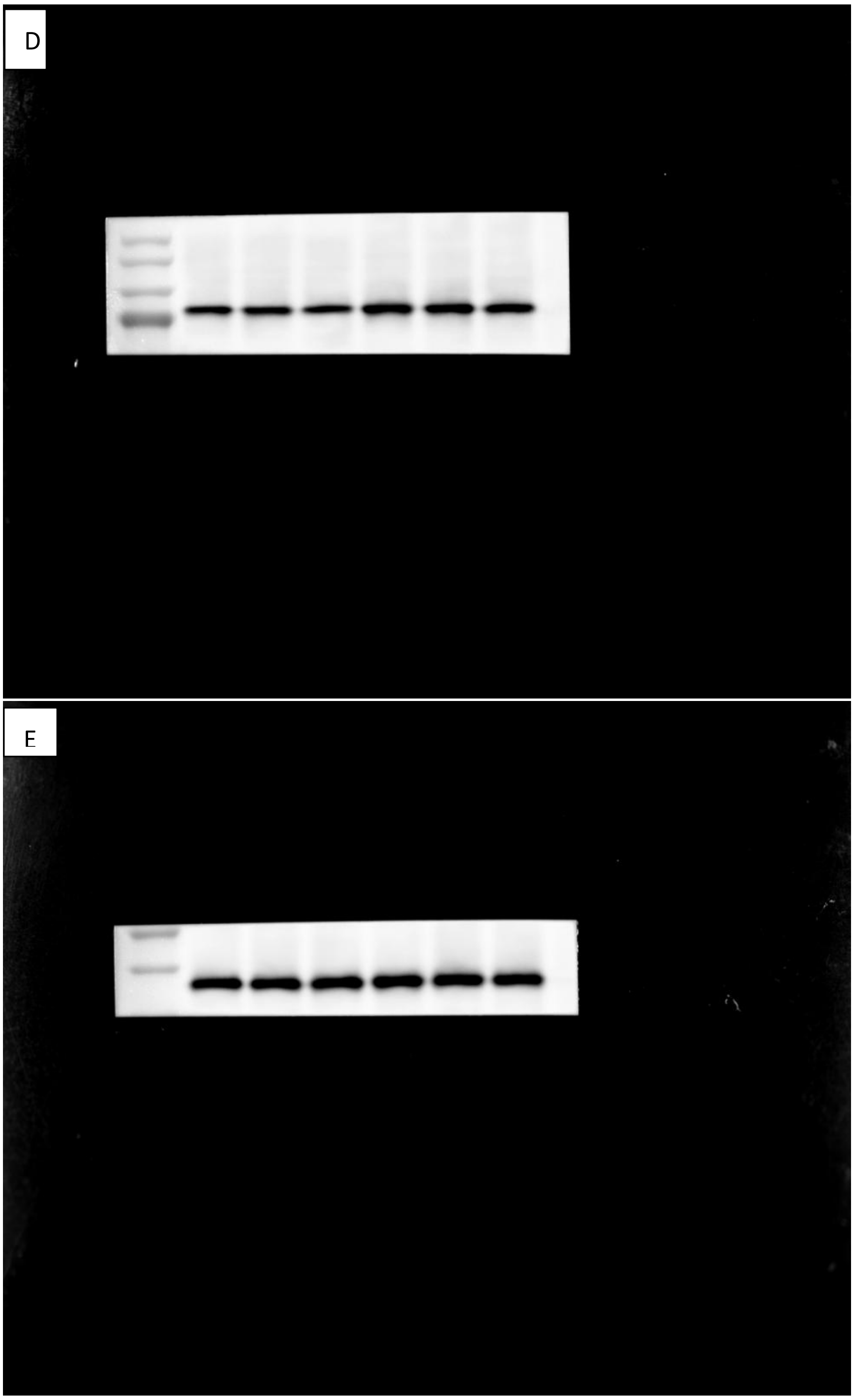

supplementary Figure10：

Figure 10A: Full-length, uncropped Western blot gel with molecular weight markers and all lanes.

Figure 10B: Cropped Western blot gel highlighting regions of interest.

Figure 10C: Western blot detection of STAR protein following overexpression.

Figure 10D: Western blot detection of FSHR protein following overexpression.

Figure 10E: Western blot detection of β-action as a housekeeping protein.

A

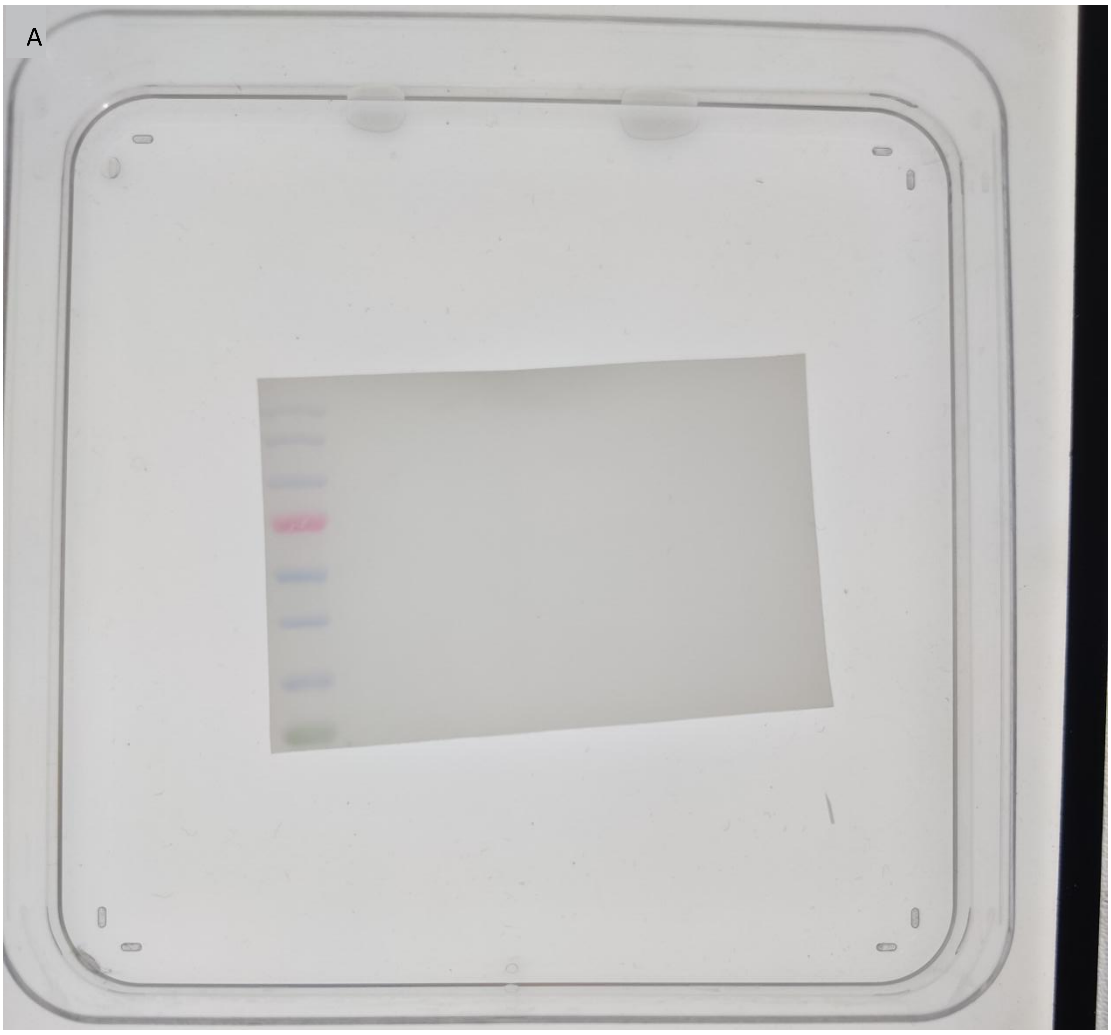

B

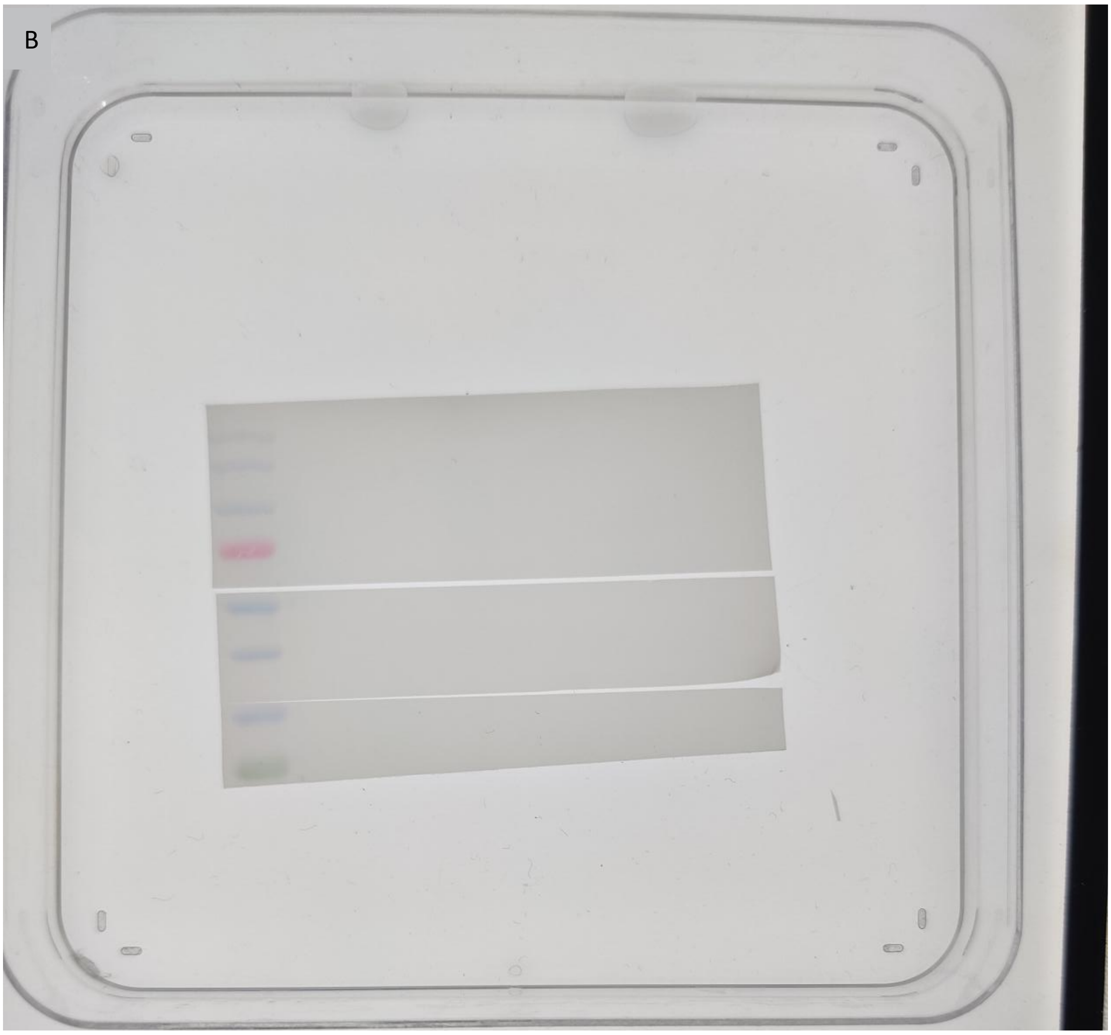

C

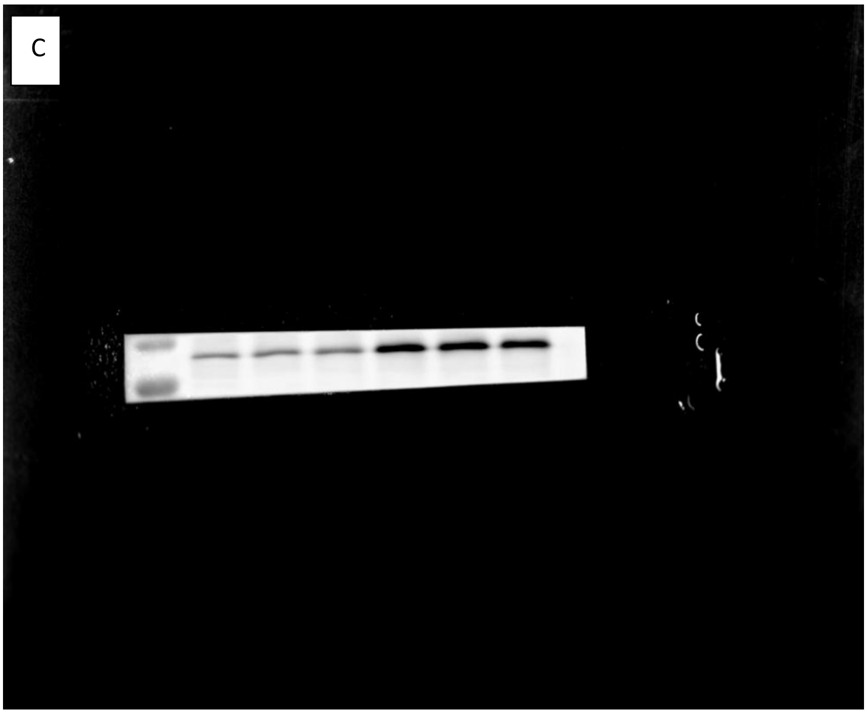

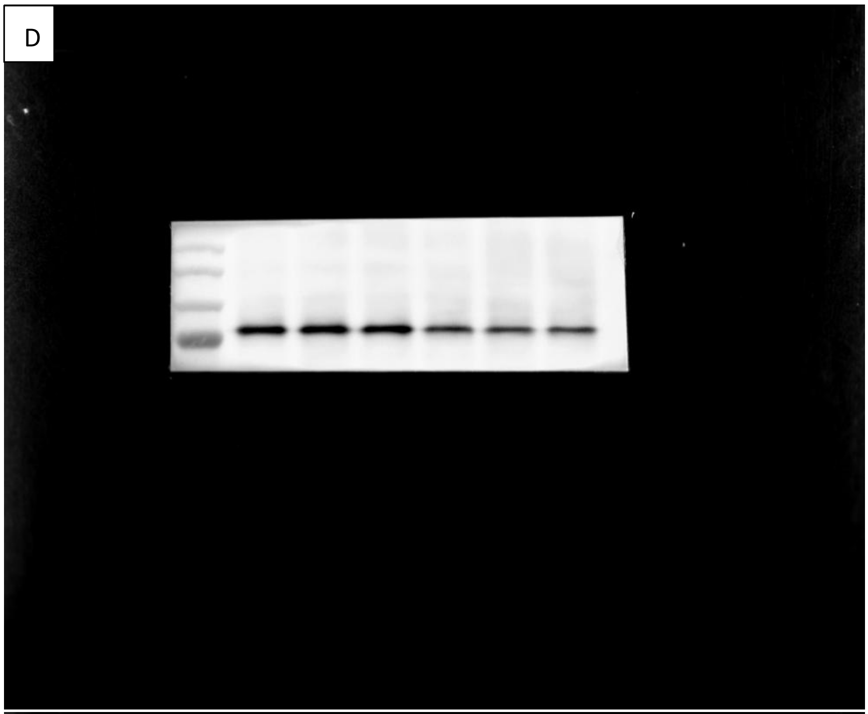

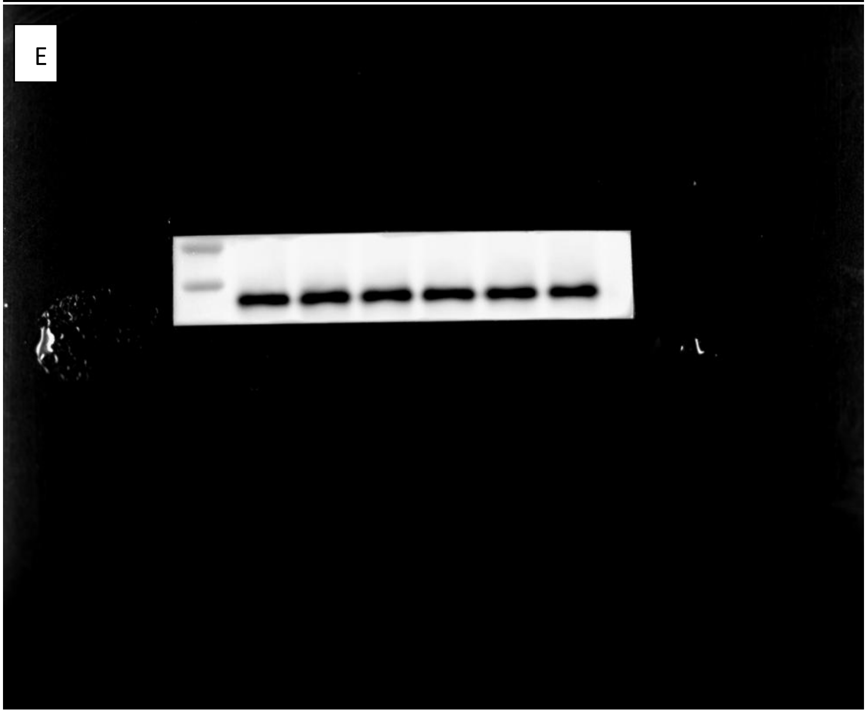

supplementary Figure11：

Figure 11A: Full-length, uncropped Western blot gel with molecular weight markers and all lanes.

Figure 11B: Cropped Western blot gel highlighting regions of interest.

Figure 11C: Western blot detection of STAR protein following interference.

Figure 11D: Western blot detection of FSHR protein following interference.

Figure 11E: Western blot detection of β-action as a housekeeping protein.

A

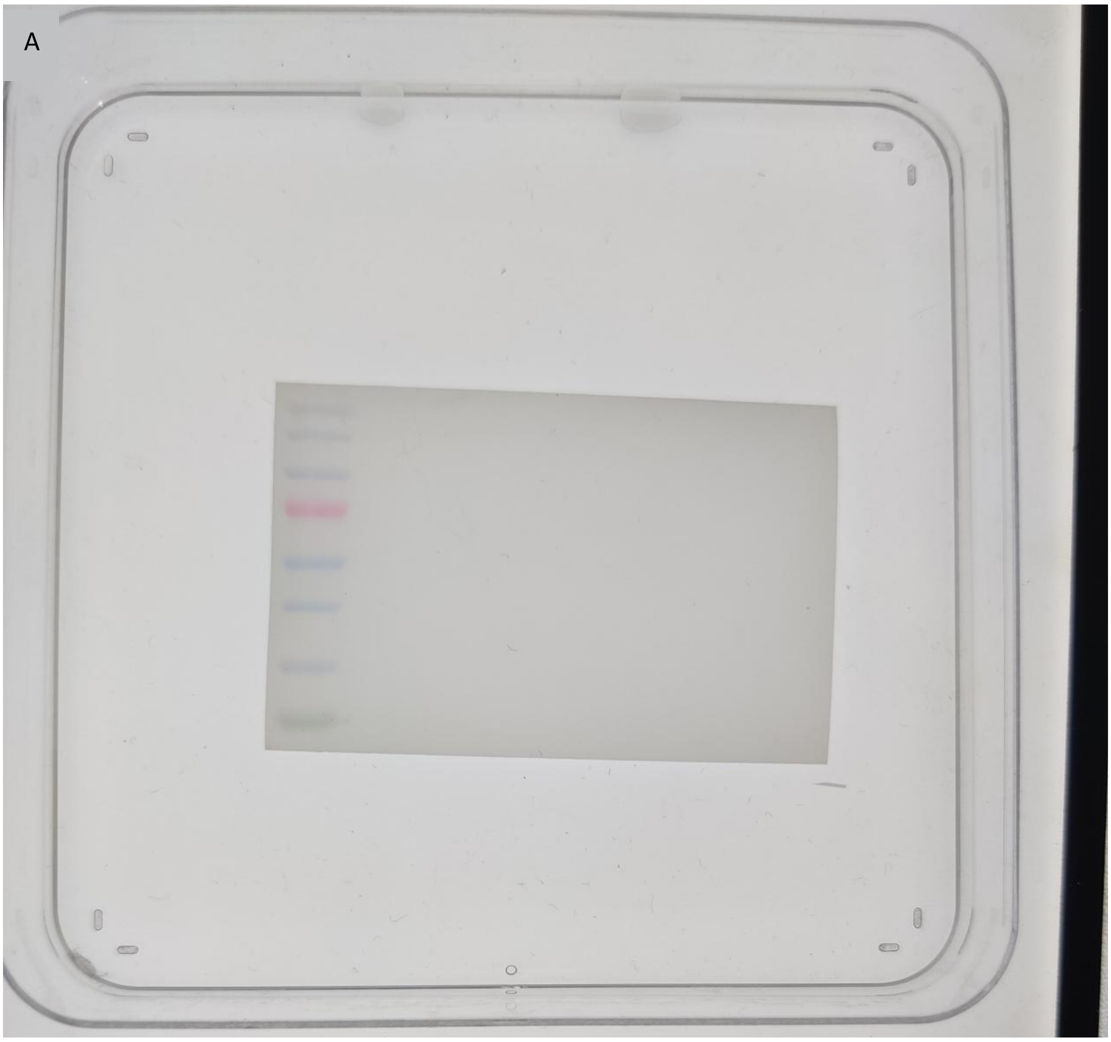

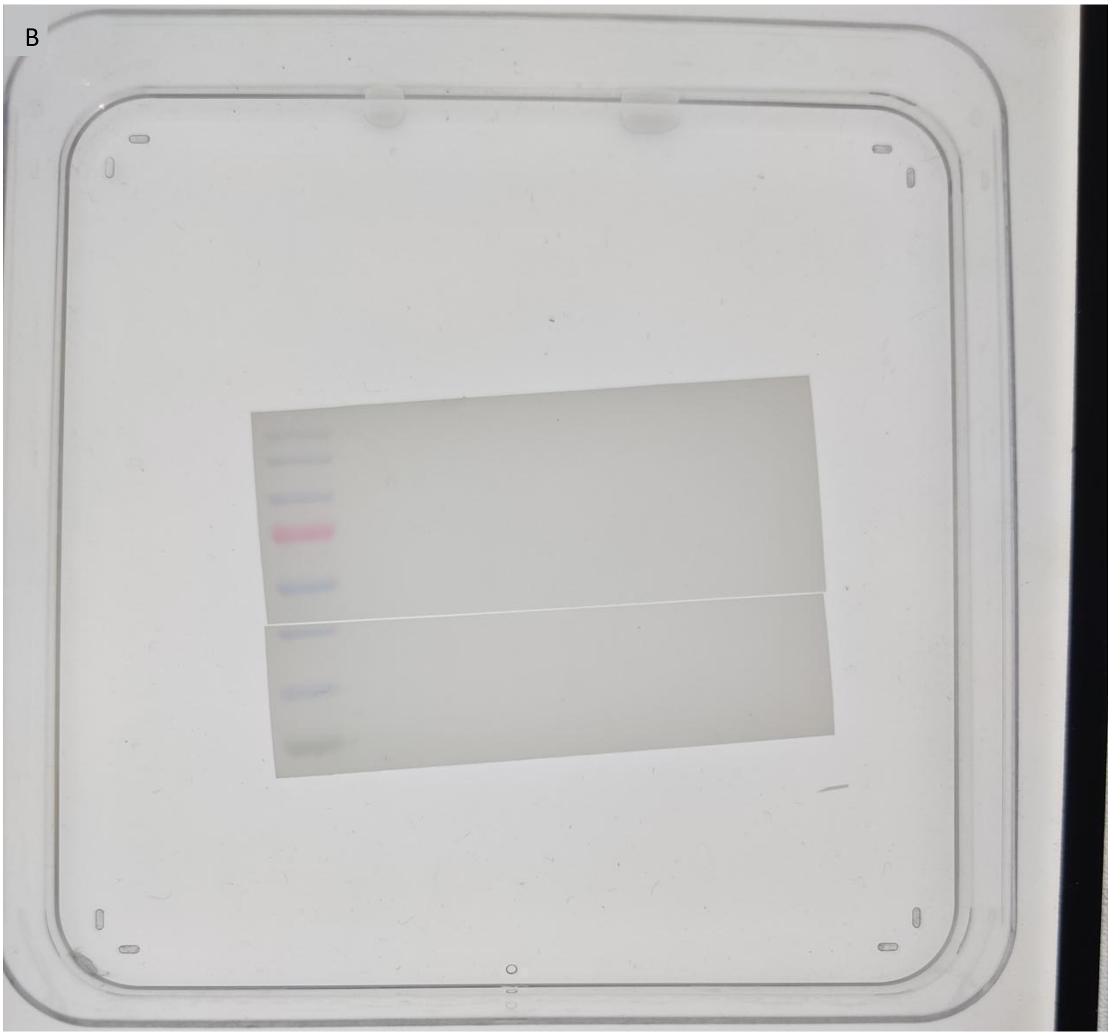
B

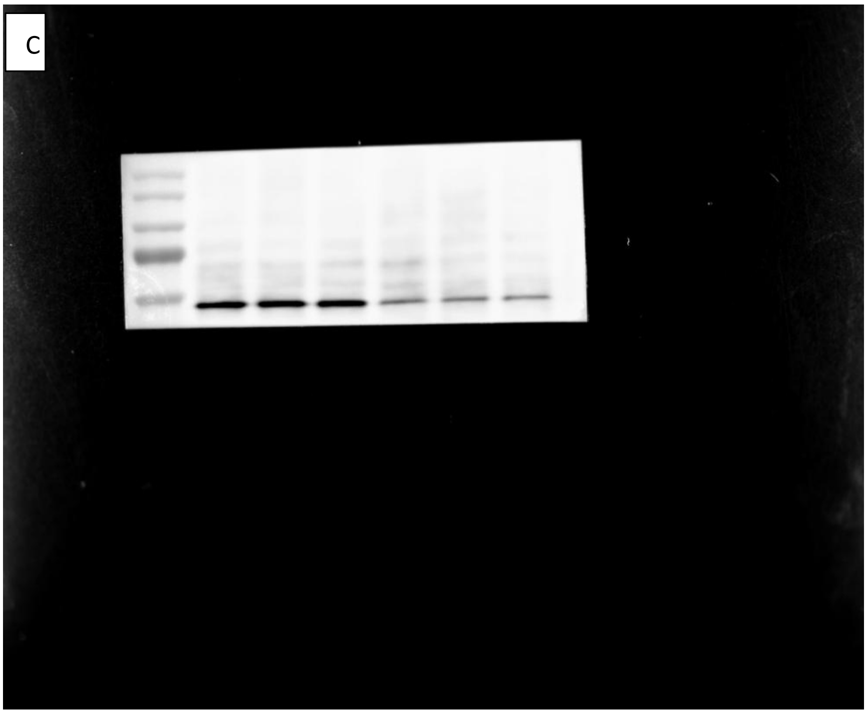
C

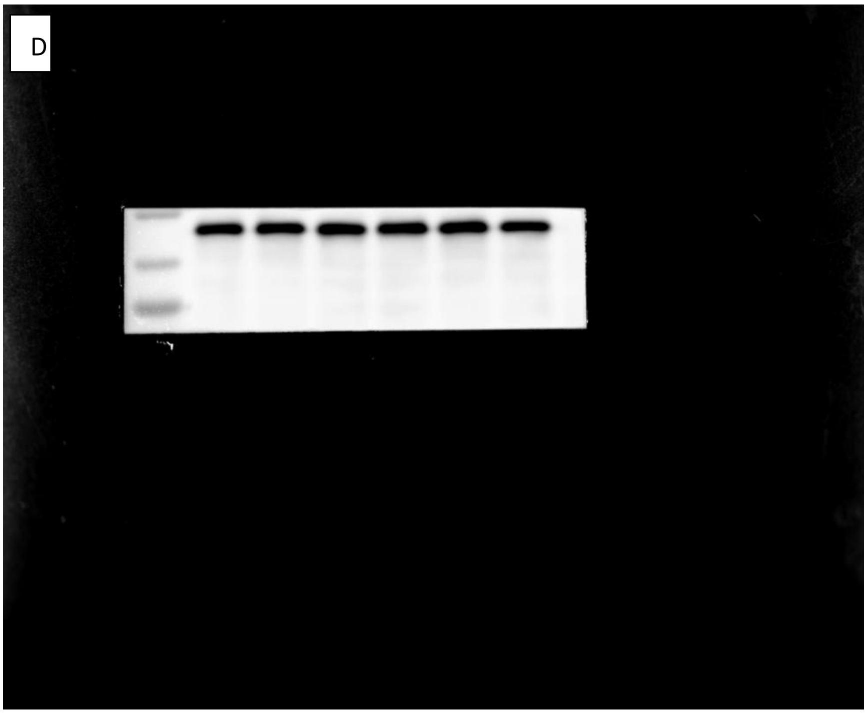

supplementary Figure12：

Figure 12A: Full-length, uncropped Western blot gel with molecular weight markers and all lanes.

Figure 12B: Cropped Western blot gel highlighting regions of interest.

Figure 12C: Western blot detection of CYP19A1 protein following overexpression.

Figure 12D: Western blot detection of β-action as a housekeeping protein.

A

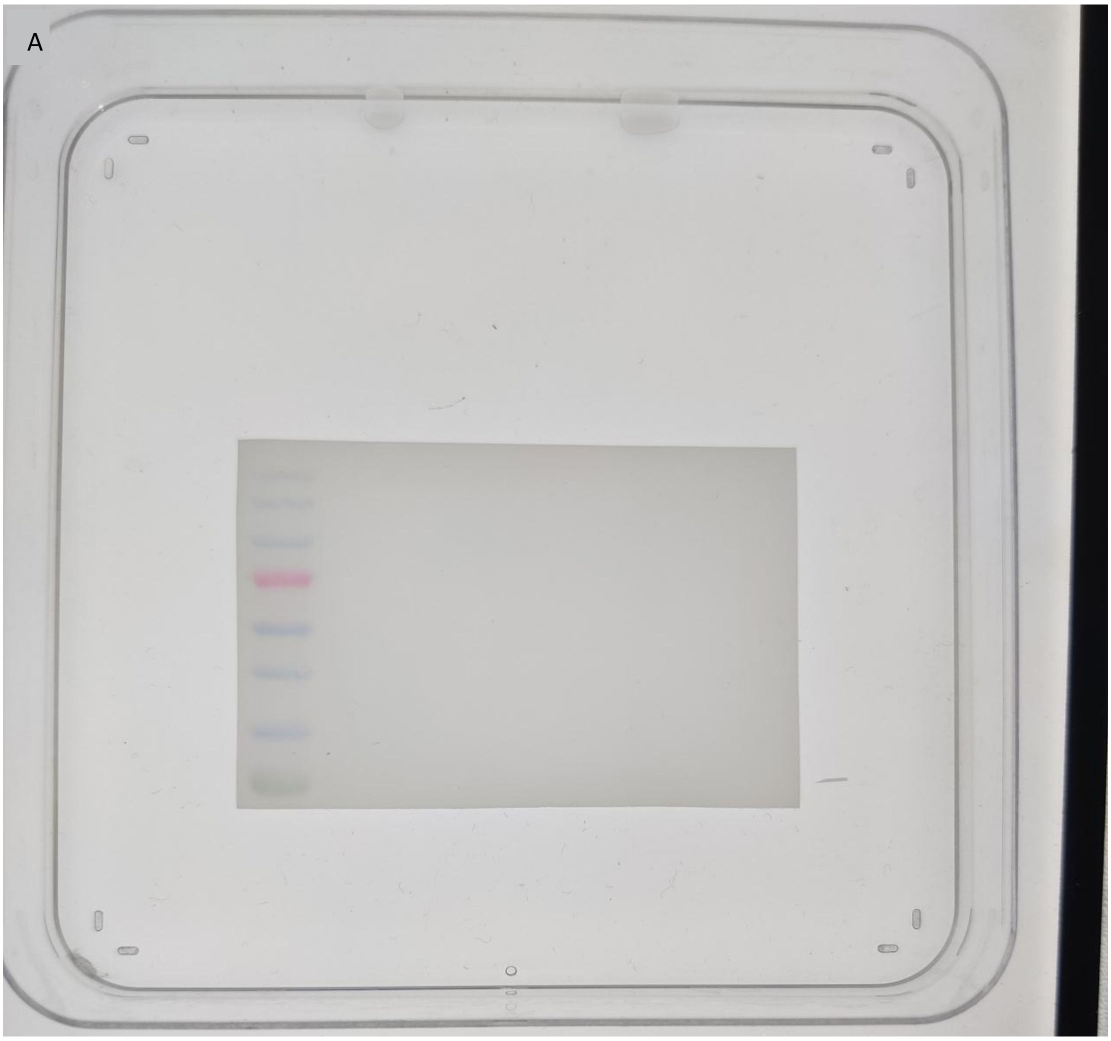

B

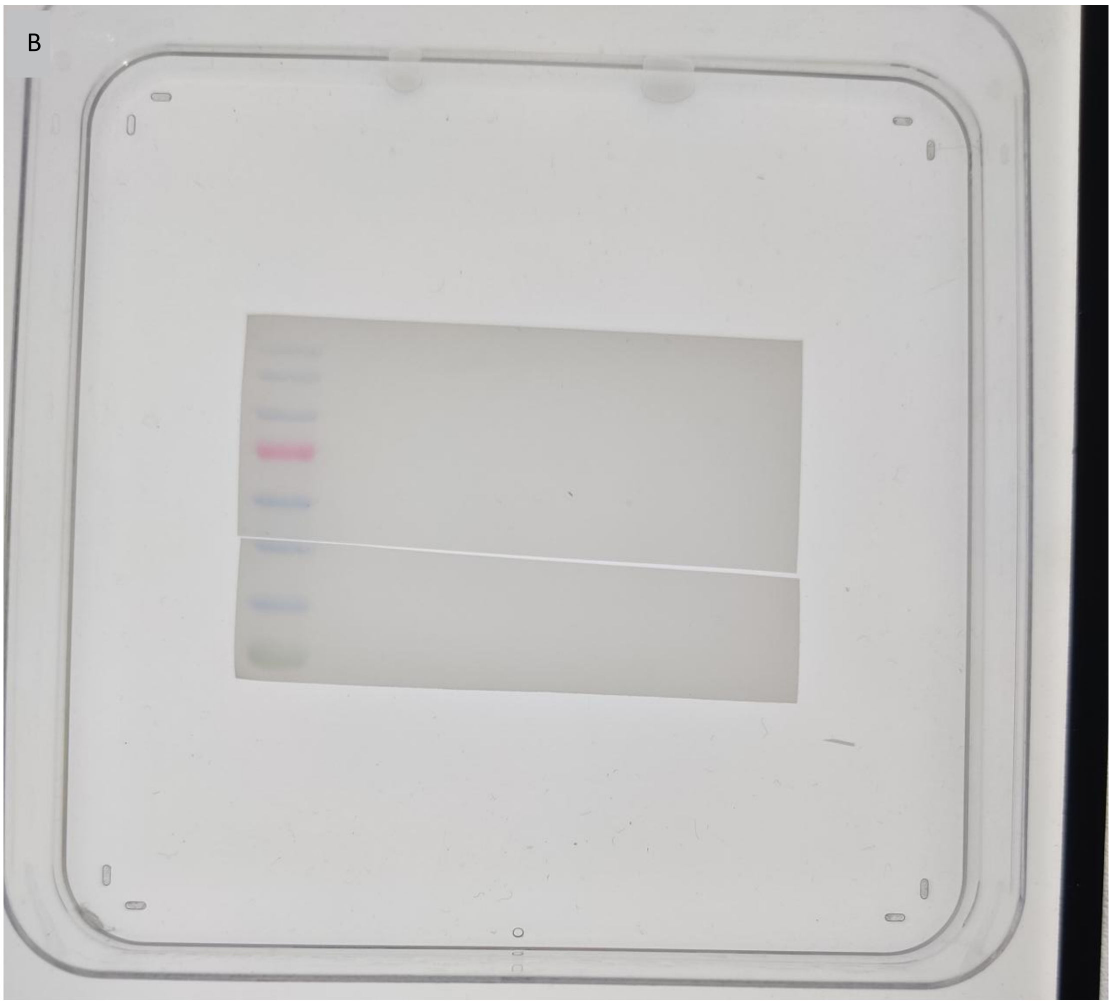

C

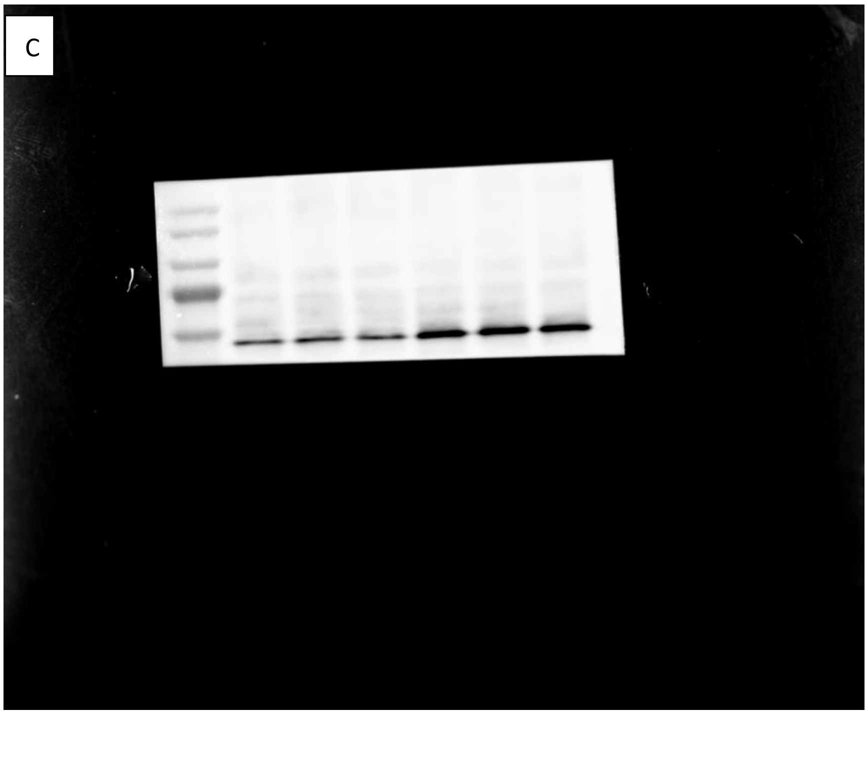

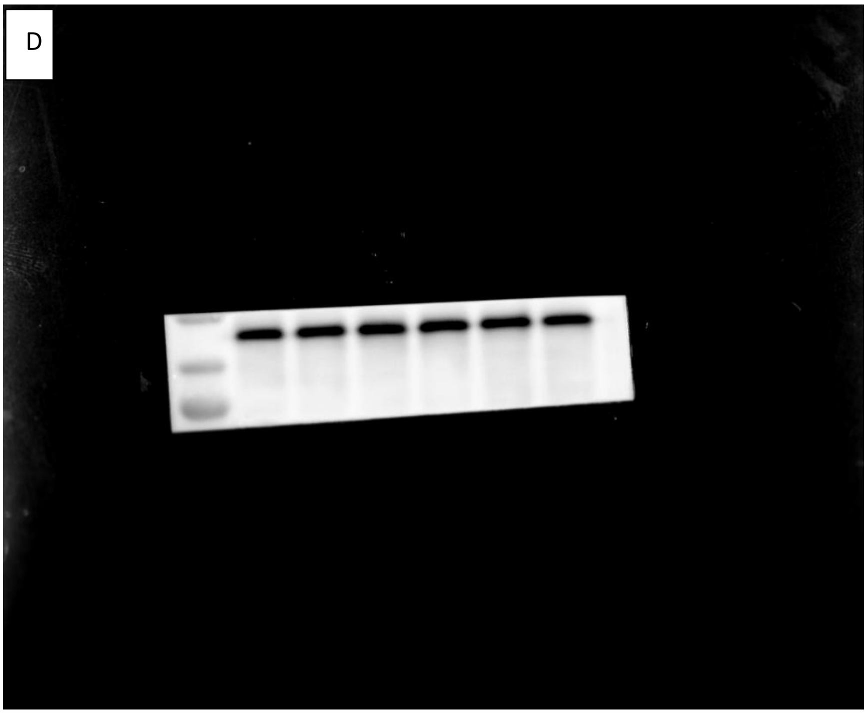

supplementary Figure13：

Figure 13A: Full-length, uncropped Western blot gel with molecular weight markers and all lanes.

Figure 13B: Cropped Western blot gel highlighting regions of interest.

Figure 13C: Western blot detection of CYP19A1 protein following interference.

Figure 13D: Western blot detection of β-action as a housekeeping protein.
